# Effect of CD14 polymorphisms on the risk of cardiovascular disease: evidence from a meta-analysis

**DOI:** 10.1186/s12944-019-1018-3

**Published:** 2019-03-28

**Authors:** Jin-Jian Xu, Ke-Qi Liu, Zhi-Min Ying, Xiao-Wei Zhu, Xue-Jin Xu, Pian-Pian Zhao, Wei-Yang Bai, Mo-Chang Qiu, Xing-Wei Zhang, Hou-Feng Zheng

**Affiliations:** 1 Diseases & Population (DaP) Geninfo Lab, School of Life Sciences, Westlake University and Westlake Institute for Advanced Study, Hangzhou, Zhejiang China; 2 Institute of Aging Research and the Affiliated Hospital, School of Medicine, Hangzhou Normal University, Hangzhou, Zhejiang China; 3 Jiangxi Medical College, Shangrao, Jiangxi China; 4 The Second Affiliated Hospital, Zhejiang University School of Medicine, Hangzhou, Zhejiang China

**Keywords:** CD14, Cardiovascular disease, Polymorphism, Meta-analysis

## Abstract

**Background:**

*CD14* polymorphisms are associated with an increased risk of cardiovascular events. So far, many studies have been conducted, whereas the results were not always consistent.

**Materials and methods:**

Twenty-six articles involving thirty-seven datasets were recruited to evaluate the association between rs2569190 (9413 patients and 7337 controls), C-159T (4813 patients and 2852 controls) polymorphisms and cardiovascular diseases in a meta-analysis. The random or fixed effect models were used to evaluate the pooled odds ratios (ORs) and their corresponding 95% confidence intervals.

**Results:**

The strongest association was observed between rs2569190 and CVD in overall population (T vs. C, OR = 1.169, 95% CI: 1.087–1.257, *p* = 2.44 × 10^− 5^). Analysis after stratification by ethnicity indicated that rs2569190 was related to CVD in East Asian population (T vs. C, OR = 1.370, 95% CI; 1.226–1.531, *p* = 2.86 × 10^− 8^) and a potential relationship in European (T vs. C, OR = 1.100, 95% CI: 1.019–1.189, *p* = 0.015). In the stratification of endpoints, the associations were found in CHD subgroup (T vs. C, OR = 1.357, 95% CI: 1.157–1.592, *p* = 2.47 × 10^− 7^) and in AMI subgroup (T vs. C, OR = 1.152, 95% CI: 1.036–1.281, *p* = 0.009). However, we did not find any association between C-159T polymorphism with cardiovascular disease under any model.

**Conclusions:**

The SNP rs2569190 significantly contribute to susceptibility and development of cardiovascular disease, particularly in the East Asian population and in the subtype CHD group, in addition, a potential association was observed in the AMI group, T allele acts as a risk factor for cardiovascular disease.

**Electronic supplementary material:**

The online version of this article (10.1186/s12944-019-1018-3) contains supplementary material, which is available to authorized users.

## Introduction

Cardiovascular disease (CVD) is a major public health problem owing to associate increased risk of human mortality [1]. By far the most common cause of acute coronary syndrome (ACS) is atherosclerosis and coronary artery stenosis, these lesions are the pathological foundation of CAD [2]. According to the number of coronary artery stenoses and the diverse clinical manifestation, CVD was defined to various clinical phenotypes (like coronary heart disease (CHD), acute myocardial infarction (AMI), myocardial infarction (MI) and so on [3]). Atherosclerosis is a process of progressive thickening and hardening of the walls of medium-sized and large arteries as a result of fat deposits on their inner lining [4]. Atherosclerosis is a pathological condition that underlies several important averse vascular events including coronary artery disease (CAD), stroke, and peripheral arterial disease, responsible for most of the cardiovascular morbidity and mortality [5]. In the 1990s, it was first time to demonstrate a strong association between inflammation and atherosclerosis, and suggested that atherosclerosis was one of chronic inflammatory diseases [5]. Recent studies have suggested that inflammation was an important factor in the initiation and development of atherosclerosis [2, 6, 7], the inflammatory reaction which in the coronary artery atherosclerosis plaque, leads to the intima damage, plaque rupture and acute cardiac ischemia [7–9]. These points revealed that infection may enhance the inflammatory processes present in atherosclerosis and CAD.

The cluster of differentiation antigen 14 (CD14) is a lipopolysaccharide (LPS) receptor located on the surface of monocytes and macrophages and it is a multiple function inflammation cytokine which is mainly produced by mature mononuclear macrophage [10]. CD14 is known as a surface marker, being glycosylated phosphatidylinositol anchored in the cell membrane (mCD14) [11]. In addition, CD14 could specially combine with LPS, transfer the activation signal to the downstream pathway through the TLR4 and bone marrow differentiation protein-2 [12, 13]. By this way, the monocytes-macrophage system was launched and many pro-inflammation cytokines as TNF-α, IL-1, IL-6 and so on were released [14]. These cytokines have multi-functions in the process of mediating initial immune response and inflammation reaction which causing the endothelia damage, disturbance of immunologic function and vascular smooth muscle cells proliferation [2]. Thus, CD14 was considered to be a key role in the process of atherosclerosis and complications.

It is generally accepted that genetic predisposition is a major risk factoring for atherosclerosis leading to CAD. In 1999, since Hubacek et al. [15] first reported that *CD14* gene single nucleotide polymorphism (SNP) rs2569190, related to the translation start site at upstream of promoter region, was apparently relevant to atherosclerosis in the Czech population, the allele T of rs2569190 was also reported being a risk factoring for MI in European population. Then, a bunch of studies were carried out to verify the causal relationship between rs2569190 and CAD in diverse ethnics. Differently, the negative results that the polymorphism was not associated with CHD and MI were observed in European participants [16]. In addition, the variant of rs2569190 whether effected CAD on Han Chinese has been a research hotpot [17, 18]. Meanwhile, a new promoter polymorphism C-159T in the gene of LPS receptor was also found to be associated with CAD by Unkelbach et al. [19]. However, the inverse finding that there was not interaction between C-159T and CAD was observed by two groups [20, 21]. Besides, the allele T of C-159T was considered to be a risk factor for CAD in a Chinese population [22], then the same conclusion was obtained in Chinese Yanbian population [23]. On the contrary, the T-to-C exchange in C-159T was considered to be risk allele in European populations, especially in older patients with a low atherosclerotic risk profile [19].

Although many studies have been conducted so far to investigate the relationship between *CD14* gene polymorphisms and CVD, the results were inconsistent. Population stratification might lead to inconsistent results, especially when allele frequency and incidence rate of the disease vary across ethnic groups. Meanwhile, inclusion of data that didn’t satisfy the requirement of meta-analysis would produce a spurious association. Therefore, in order to reduce the limitations of single study and to overcome the possible random errors, a large-scale meta-analysis involving multifarious ethnics was performed by us.

## Materials and methods

### Identification of eligible studies

To analyze the association between *CD14* gene (SNP rs2569190, C-159T) and CVD, all published literature before December 2017 that researched the relationship between these polymorphisms and CVD risk were concluded. The electronic databases were used including PubMed databases (National Center for Biotechnology, National Library of Medicine), CNKI (China National Knowledge Infrastructure), and Web of Science were retrieved by using the keywords “CD14”, “C-260T”, “C-159T”, “rs2569190”, “polymorphism” connect to “CVD”, “coronary artery disease”, “coronary heart disease”, “myocardial infarction”, or “atherosclerosis” without language restrictions. Finally, we extracted data from the published articles, not included meetings or any conference abstracts. All of the included studies used either case-control or nested case-control design. Appropriate diagnosis criteria (e.g., arteriography confirmed; changes of electrocardiographic and clinical symptoms according to the WHO criteria; a documented history of coronary intervention) and proper genotyping methods were used in most of the studies.

### Selection criteria and data extraction

Such major criteria must be followed for included studies (1) Original papers containing complete data, (2) Case–control or cohort studies that assessed the association of CD14 gene -159C/T and rs2569190 polymorphisms with CVD, and (3) Sufficient data to calculate the odds ratio (OR) or *P* value, (4) Relevant cardiovascular outcomes were angiographically confirmed (generally by WHO criteria) and coronary stenosis was diagnosed as at least one coronary artery stenosis (no less than 50% by a coronary angiography [24, 25]), (5) The genotype distribution in the control group for each individual study should follow Hardy-Weinberg equilibrium (HWE).

The primary reasons for excluded studies: (1) Case-only studies, review or meta-analysis articles, (2) Deviate from the major selection criteria, (3) Overlapping or that supplied inadequate data, (4) Repeated publications or the same authors employed similar data in different papers, the data was only used once.

The study data was extracted based on standard protocol. For studies in which data could not be separated according to type of CVD from published data, cases were classified in the more inclusive category of coronary stenosis for the purposes of subsidiary analyses. Disagreement was settled by a consensus between all authors. Where essential information was not presented in articles, every effort was made to contact the authors. All procedures conformed to the guidelines for meta-analysis of observational studies in epidemiology. The following information were extracted independently by individual in our study: first author, year of publication, ethnicity, study design, types of CVD endpoints, HWE status among control, sample size of case and control, number of genotype and allele frequency.

### Statistical analysis

We calculated the allele frequency for each study in allele counting method, the Hardy–Weinberg equilibrium (HWE) was tested by using the Chi square test [26]. We employed pooled ORs and 95% confidence intervals (CIs) to evaluate the strength of association between polymorphisms and cardiovascular disease for every eligible study [27]. The methodology of Cochran’s Q-statistic was used to evaluate the heterogeneity, which similar to the previous study in our lab [28]. If the *P* value in heterogeneity test was higher than 0.1, the fixed effect model was used. Moreover, the random effect model was used [29–31]. We used the following formula to quantified the effect of heterogeneity :*I*^2^ = 100 %  × (*Q* − *dƒ* )/*Q*. The proportion of between-study variability attributable to heterogeneity was indicated by *I*^2^ value, and *I*^2^ values of 25, 50 and 75% were considered to be of low, moderate and high heterogeneity, respectively. If study groups revealed no heterogeneity, the similar results were produced in fixed and random effects models and, otherwise the random effects model usually produced wider CIs than the fixed effects model. In this meta-analysis, *P* value of less than 0.05 was considered a statistically significant.

In order to get exacting search results, we evaluated possible publication bias by Egger’s linear regression text [32]. If *P* value < 0.05 the statistical publication bias was considered [33]. Moreover the Begg’s test also used a funnel plot to evaluate the publication bias [32]. For sensitivity analysis, we removed one study orderly from the total and tested residual studies [34]. Statistical analysis was carried out using the software program STATA12.0 (Stata Corporation, College Station, Texas).

## Results

### Studies included in the meta-analysis

**Fig. 1:**
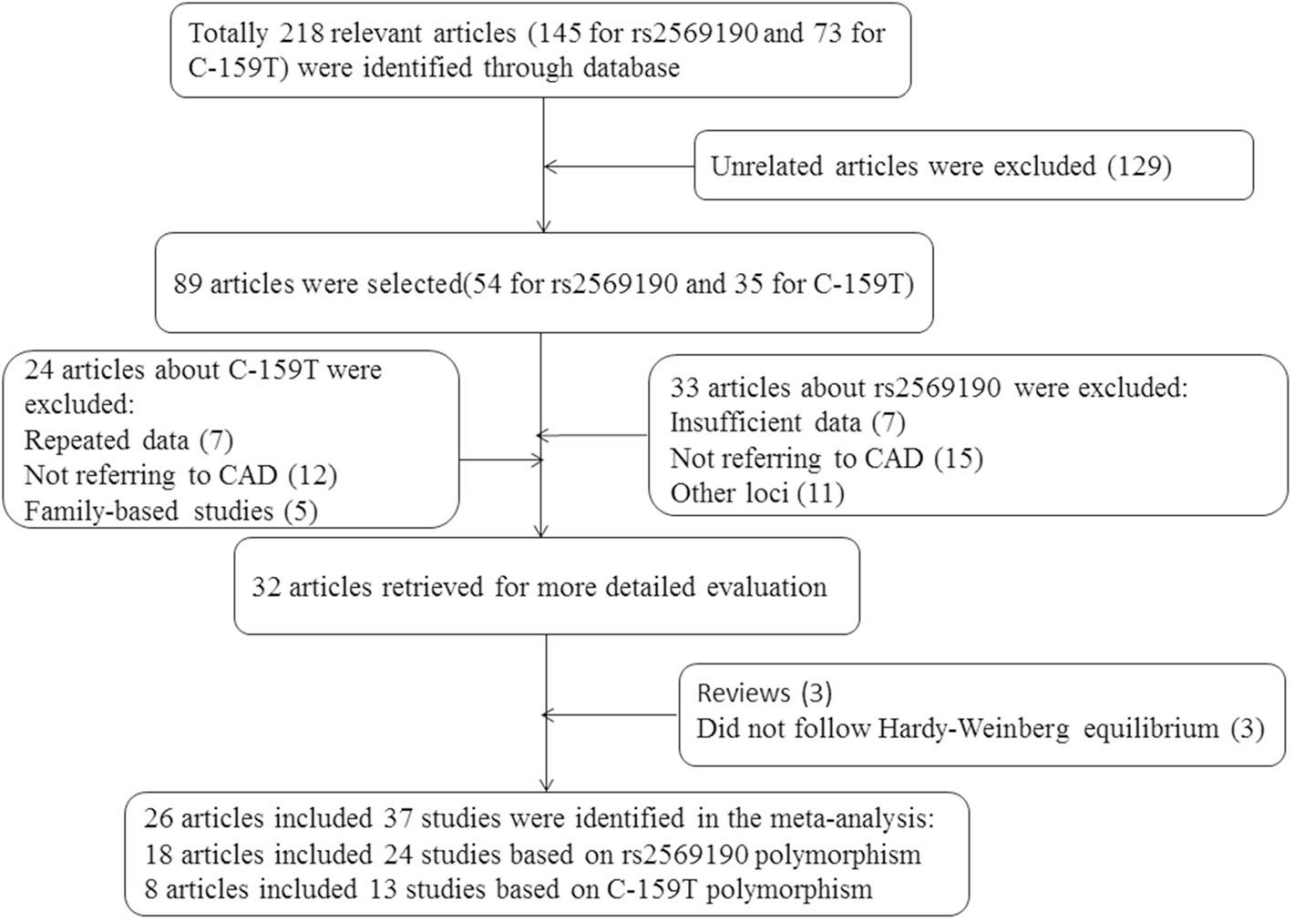
The process of the articles selected in this meta-analysis

In this meta-analysis, totally 218 relevant articles (145 for rs2569190 and 73 for C-159T) were searched. After reading titles and abstracts, we excluded irrelevant studies, 89 articles (54 for rs2569190 and 35 for C-159T) for further reading. Then, we excluded 57 articles (33 for rs2569190 and 24 for C-159T), because of no data, insufficient data, repeated date, family-based studies and not referring to cardiovascular disease (CVD). Thus, 32 articles (23 for rs2569190 and 9 for C-159T) met the study inclusion criteria. Lastly, after excluding three groups of data in which the control populations deviated from HWE [17, 35, 36] and three reviews [37–39] about rs2569190 and C-159T. After filtering, 26 eligible studies involved 37 data sets were finally included [15, 16, 18–23, 40–55], in which two articles contained dual data (which articles included two type data about rs2569190 and C-159T) [19, 40]. Eventually, 26 studies providing 14,226 cases and 10,189 controls (rs2569190: 9413 patients and 7337 controls; C-159T: 4813 patients and 2852 controls) were pooled to evaluate the relationship between SNPs of CD14 and CVD in the meta-analysis (Table 1). The flowchart of selecting article process is presented in Fig. 1.

**Table 1:** The basic information of every studies included in this meta-analysis

Polymorphisms	Study	Year	Ethnicity	design	Endpoint	P(HWE)	Sample size		Genotypes						Allele frequencies (%)			
							Cases	Controls	Cases			Controls			Cases		Controls	
									CC	CT	TT	CC	CT	TT	C	T	C	T
rs2569190 (C-260T)	Wei et al. [40]	2006	East Asian	CC	CHD	0.22	246	258	58	112	76	83	118	57	46.3	53.7	55.0	45.0
	Zhang et al. [18]	2006	East Asian	CC	CHD	0.05	193	225	20	95	78	39	92	94	35.0	65.0	37.8	62.2
	Li et al. [41]	2007	East Asian	CC	CHD	0.14	193	225	29	95	69	47	124	54	40.0	60.0	48.0	52.0
	Xie et al. [42]	2008	East Asian	CC	CHD	0.24	241	149	49	127	65	56	65	28	46.7	53.3	59.4	40.6
	Liu et al. [55]	2010	East Asian	CC	AMI	0.19	12O	130	23	56	41	43	57	30	42.5	57.5	55.0	45.0
	Shimada et al. [43]	2000	East Asian	CC	MI	0.83	128	83	27	49	52	21	43	19	40.0	60.0	51.0	49.0
	Hohda et al. [44]	2003	East Asian	CC	MI	0.2	502	527	97	242	163	115	278	134	43.4	56.6	48.2	51.8
	Nauck et al. [16]	2002	European	NCC	AMI	0.43	2559	697	675	1262	622	188	358	151	51.0	49.0	53.0	47.0
	Nauck et al. [16]	2002	European	NCC	MI	0.43	1599	697	444	758	397	188	358	151	51.0	49.0	53.0	47.0
	Unkelbach et al. [19]	1999	European	CC	MI	0.99	1053	1175	292	520	241	339	584	252	52.0	48.0	54.0	46.0
	Koenig et al. [45]	2002	European	CC	AMI	0.62	312	476	75	164	73	126	243	107	50.3	49.7	52.0	48.0
	Longobardo et al. [46]	2003	European	CC	AMI	0.38	215	215	44	101	70	55	101	59	44.0	56.0	49.0	51.0
	Morange et al. [47]	2005	European	CC	MI	0.38	194	197	42	98	54	39	104	54	47.0	53.0	46.0	54.0
	Giacconi et al. [48]	2007	European	CC	CHD	0.14	146	148	39	69	38	48	80	20	50.4	49.6	59.0	41.0
	Hubacek et al. [15]	1999	European	CC	MI	0.11	178	135	52	77	49	61	53	21	50.8	49.2	64.8	35.2
	Lorenzova et al. [49]	2007	European	CC	AMI	0.32	230	562	63	116	51	166	268	128	53.0	47.0	53.0	47.0
	Arroyo-Espliguro [50]	2005	European	CC	AMI	0.35	194	94	45	85	64	31	42	21	45.1	54.9	55.3	44.7
	Arroyo-Espliguro [50]	2005	European	CC	CHD	0.35	140	94	34	78	28	31	42	21	52.1	47.9	55.3	44.7
	Morange et al. [47]	2005	European	CC	MI	0.41	54	70	12	28	14	24	31	15	48.0	52.0	56.0	44.0
	Morange et al. [47]	2005	European	CC	MI	0.19	99	121	20	57	22	29	53	39	49.0	51.0	46.0	54.0
	Morange et al. [51]	2004	European	NCC	MI	0.09	128	253	43	59	26	69	113	71	57.0	43.0	50.0	50.0
	Morange et al. [51]	2004	European	NCC	CHD	0.75	123	243	31	58	34	61	124	58	49.0	51.0	51.0	49.0
	Morange et al. [47]	2005	European	CC	MI	0.2	179	176	60	94	25	65	77	34	60.0	40.0	59.0	41.0
	Zee et al. [52]	2001	America	NCC	MI	0.99	387	387	98	215	74	108	193	86	53.0	47.0	53.0	47.0
C-159T	Jin et al. [23]	2016	East Asian	CC	EH-LVH	0.99	116	108	27	58	31	25	54	29	48.3	51.7	48.3	51.7
	Jin et al. [23]	2016	East Asian	CC	EH-NLVH	0.99	107	108	24	46	37	25	54	29	43.8	56.2	48.3	51.7
	Jin et al. [23]	2016	East Asian	CC	EH-LVH	1	103	108	23	52	28	26	54	28	47.6	52.4	49.1	50.9
	Jin et al. [23]	2016	East Asian	CC	EH-NLVH	1	100	108	23	44	33	26	54	28	45.0	55.0	49.1	50.9
	Wei et al. [40]	2006	East Asian	CC	CHD	0.09	246	258	47	128	71	39	139	80	45.1	54.9	42.1	57.9
	Li et al. [22]	2005	East Asian	CC	CHD	0.2	162	196	24	75	63	54	89	53	38.0	62.0	59.2	40.8
	Haberbosch et al. [21]	2009	European	CC	AMI	0.46	54	252	16	24	14	71	131	50	51.9	48.1	54.2	45.8
	Haberbosch et al. [21]	2009	European	CC	MI	0.46	146	252	50	69	27	71	131	50	57.9	42.1	54.2	45.8
	Koch et al. [20]	2002	European	CC	AMI	0.43	998	340	273	498	227	88	177	75	52.0	48.0	52.0	48.0
	Koch et al. [20]	2002	European	CC	MI	0.43	793	340	232	390	171	88	177	75	54.0	46.0	52.0	48.0
	Unkelbach et al. [19]	1999	European	CC	AMI	0.36	1727	501	491	864	372	140	240	121	53.0	47.0	52.0	48.0
	Damiano et al. [53]	2012	European	CC	CHD	0.07	51	49	7	23	21	13	18	18	36.0	64.0	44.0	56.0
	Banerjee et al. [54]	2009	Indian	CC	AMI	0.14	210	232	45	116	49	38	126	68	49.0	51.0	44.0	56.0

*CHD* coronary heart disease, *AMI* acute myocardial infarction, *MI* myocardial infarction, *EH –LVH/ EH –LVH* essential hypertension with left/not left ventricular hypertrophy, *CC* case–control, *NCC* nested case–control, *Endpoint* diseases of cases, *HWE* Hardy-Weinberg Equilibrium

### Meta-analysis results

#### SNP rs2569190 and cardiovascular disease risk

**Fig. 2:**
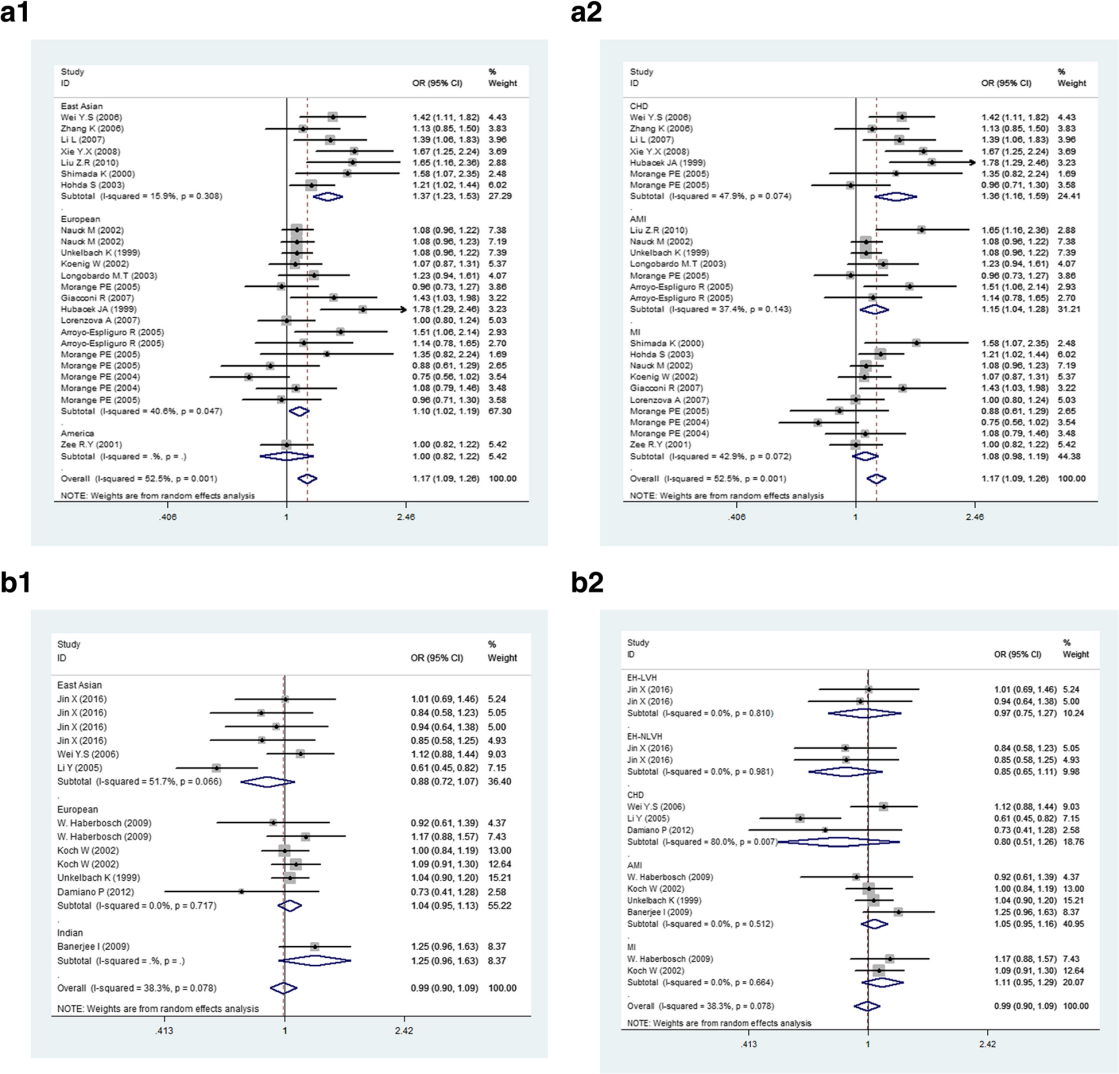
Forest plot for the meta-analysis of the association between CD14 gene polymorphisms and CVD. **a1** rs2569190 and CVD (T VS.C), stratification by ethnicity. **a2** rs2569190 and CVD (T VS.C), stratification by endpoint. **b1** C-159T and CVD (T VS.C), stratification by ethnicity. **b2** C-159T and CVD (T VS.C), stratification by endpoint

The test of heterogeneity indicated that there was potential heterogeneity in the overall population (*p* = 0.001, *I*^2^ = 52.5%), but after stratified by ethnicity, the heterogeneity was resolved in any subgroup (*p* = 0.308, *I*^2^ = 15.9%). An outstanding association was found in the overall population, under the random effect model (T vs. C, OR = 1.169, 95% CI: 1.087–1.257, *P* = 2.44 × 10^− 5^, Table 2, Fig. 2a1). Accordingly, dominant and recessive models were also tested to estimate the relationship between rs2569190 and CVD risk, the significant associations were observed in overall population (TT + CT vs. CC, OR = 1.233, 95% CI: 1.110–1.370, *p* = 3.79 × 10^− 5^, Table 2, Additional file 1: Figure S1a; TT vs. CC + CT, OR = 1.195, 95% CI: 1.062–1.345, *p* = 0.003, Table 2, Additional file 1: Figure S1b). In the subgroup’s analysis by ethnicity, we obtained the most significant relationship between rs2569190 with CVD, particularly in East Asian population under allele model (T vs. C, OR = 1.370, 95% CI: 1.226–1.531, *p* = 2.86 × 10^− 8^, Table 2, Fig. 2a1), dominant model (TT + TC vs. CC, OR = 1.574, 95% CI: 1.278–1.938, *p* = 9.11 × 10^− 7^, Table 2, Additional file 1: Figure S1a) and recessive model (TT vs. TC + CC, OR = 1.492, 95% CI: 1.236–1.801, *p* = 3.05 × 10^− 5^, Table 2, Additional file 1: Figure S1b). Besides, the results revealed that rs2569190 was associated with CVD in European under allele and dominant models (T vs. C, OR = 1.100, 95% CI: 1.019–1.189, *p* = 0.015, Table 2, Additional file 1: Figure S1a; TT + TC vs. CC, OR = 1.113, 95% CI: 1.006–1.232, *p* = 0.039, Table 2, Additional file 1: Figure S1b). Meanwhile, no relationship under any model was found in American population (Table 2, Fig. 2a1).

**Table 2:** Meta-analysis of the association between rs2569190 (C-260T) polymorphism and CVD risk

Sub-group analysis	No of data sets	No of cases/controls		Allele model (T VS.C)		Dominant model (CC VS.TT + CT)				Recessive model (TT VS.CC + CT)		
		Cases	Controls	OR (95% CI)	POR	PH	OR (95% CI)	POR	PH	OR (95% CI)	POR	PH
Overall	24	9413	7337	1.169(1.087–1.257)	2.44 × 10^−5*^	0.001	1.233(1.110–1.370)	3.79 × 10^−5*^	0.02	1.195(1.062–1.345)	0.003*	0.002
Ethnicity												
East Asian	7	1503	1597	1.370(1.226–1.531)	2.86 × 10^−8*^	0.308	1.574(1.278–1.938)	9.11 × 10^−7*^	0.217	1.492(1.236–1.801)	3.05 × 10^−5^*	0.22
European	16	7403	5353	1.100(1.019–1.189)	0.015	0.047	1.113(1.006–1.232)	0.039	0.237	1.112(0.975–1.269)	0.113	0.04
America	1	387	387	1.000(0.819–1.221)	1	–	1.142(0.830–1.571)	0.416	–	0.827(0.584–1.173)	0.287	–
Endpoint												
CHD	7	1019	1005	1.357(1.157–1.592)	2.47 × 10^−7*^	0.074	1.669(1.388–2.007)	2.48 × 10^−6*^	0.433	1.342(1.011–1.781)	0.042	0.035
AMI	7	762	1244	1.152(1.036–1.281)	0.009	0.143	1.186(0.997–1.412)	0.054	0.131	1.162(1.030–1.310)	0.015	0.483
MI	10	4641	3915	1.077(0.976–1.188)	0.139	0.072	1.061(0.949–1.186)	0.297	0.799	1.118(0.907–1.378)	0.297	0.002

*OR* odd ratio, *95%CI* 95% confidence interval, *P*_*OR*_*P* value for the test of association, *P*_*H*_*P* value for heterogeneity analysis

^∗^Significant *P*-value

Similarly, we carried out subgroup analysis by endpoints to estimate the relationship between rs2569190 and the specific isoforms of CVD, a very significant association was identified between rs2569190 and CHD under allelic model (T vs. C, OR = 1.357, 95% CI: 1.157–1.592, *p* = 2.47 × 10^− 7^, Table 2, Fig. 2a2), dominant model (TT + TC vs. CC, OR = 1.669, 95% CI: 1.388–2.007, *p* = 2.48 × 10^− 6^, Table 2, Additional file 1: Figure S2a) and a potential association in recessive model (TT vs. CC + CT, OR = 1.342, 95% CI: 1.011–1.781, *p* = 0.042, Table 2, Additional file 1: Figure S2b). Besides, the association between rs2569190 and AMI was also observed under allelic model (T vs. C, OR = 1.152, 95% CI: 1.036–1.281, *p* = 0.009, Table 2, Fig. 2a2), and recessive model (TT vs. CC + CT, OR = 1.162, 95% CI: 1.030–1.310, *p* = 0.015, Table 2, Additional file 1: Figure S2b).

#### C (− 159) T and cardiovascular disease risk

The association between C-159T and CVD was conducted in 13 independent studies with 7665 participants in this meta-analysis and all of datasets followed the inclusion criteria. The test of heterogeneity in the overall population was not significant (*p* = 0.324, *I*^2^ = 12.1%), suggesting that the fixed effect model could be used.

We didn’t observe association in overall population under allele model (T vs. C, OR = 1.009, 95% CI: 0.941–1.082, *p* = 0.854, Table 3, Fig. 2b1). We also tested the dominant and recessive genetic models in overall population, but no associations were found in the two models (Table 3, Additional file 1: Figure S1a, d). In the subgroup analysis by ethnicity, no potential association was found between C-159T and the risk of CVD in any populations under all genetic models (Table 3, Fig. 2b1, Additional file 1: Figure S1c, d). Furthermore, we also performed subgroup analysis by endpoints in three models, respectively, which indicated that no statistically significant association was discovered in these isoforms of CVD (Table 3, Fig. 2b2, Additional file 1: Figure S2c, d). In essence, the results did not reveal any allele-specific or genotype-specific relation of the C-159T with CVD (Table 3).

**Table 3:** Meta-analysis of the association between C-159T polymorphism and CVD risk

Sub-group analysis	No of data sets	No of cases/controls		Allele model (T VS.C)			Dominant model (CC VS.TT + CT)			Recessive model (TT VS.CC + CT)		
		Cases	Controls	OR (^b^95% CI)	POR	PH	OR (95% CI)	POR	PH	OR (95% CI)	POR	PH
Overall	13	4813	2852	1.009(0.941–1.082)	0.854	0.078	1.049(0.936–1.176)	0.41	0.164	1.009(0.900–1.132)	0.878	0.324
Ethnicity												
East Asian	6	834	886	0.881(0.723–1.075)	0.212	0.066	1.120(0.888–1.413)	0.34	0.109	1.198(0.974–1.474)	0.087	0.327
European	6	3769	1734	1.039(0.953–1.132)	0.387	0.717	0.923(0.806–1.058)	0.249	0.488	0.962(0.832–1.113)	0.602	0.751
Indian	1	210	232	1.249(0.958–1.628)	0.101	–	0.718(0.445–1.160)	0.176	–	0.734(0.479–1.125)	0.156	–
Endpoint												
EH-LVH	2	219	216	0.974(0.746–1.271)	0.845	0.81	1.045(0.669–1.631)	0.847	0.817	1.028(0.672–1.573)	0.898	0.87
EH-NLVH	2	207	216	0.846(0.646–1.109)	0.227	0.981	1.051(0.669–1.653)	0.828	0.967	1.424(0.938–2.162)	0.097	0.957
CHD	3	459	503	0.804(0.514–1.257)	0.338	0.007	1.292(0.953–1.785)	0.121	0.007	1.186(0.904–1.556)	0.217	0.1
AMI	4	2989	1325	1.048(0.950–1.156)	0.351	0.512	0.926(0.790–1.085)	0.342	0.729	0.917(0.778–1.080)	0.3	0.314
MI	2	939	592	1.109(0.952–1.293)	0.184	0.664	0.817(0.642–1.038)	0.098	0.669	0.957(0.735–1.246)	0.743	0.851

*OR* odd ratio, *95%CI* 95% confidence interval, *P*_*OR*_*P* value for the test of association, *P*_*H*_*P* value for heterogeneity analysis

#### Allele frequency of the rs2569190 and comparing to the 1000 genome phase 3 population

We displayed the alleles frequencies of different ethnicities in our meta-analysis and 1000 genomes alleles frequencies of rs2569190 in Table 4. In view of the sample size and population, the allelic frequencies of rs2569190 in this meta-analysis were consistent with the allelic frequencies in the 1000 Genome Project EAS (East Asian ancestry), EUR (European ancestry), respectively, however, there was distinction between the allele frequencies in AMR (Admixed American) and 1000 Genomes Project. Although such inconsistent allele frequencies in AMR were observed in the comparison to 1000 Genomes Project, the approximate allele frequency was obtained in overall population to 1000 Genomes Project. Because of the deficient data, we fail to make a comparison for C-159T to1000 Genomes Project (Table 4).

**Table 4:** The allele frequency comparison between the meta-analysis and 1000 Genomes Project

Polymorphism	Populations	Meta-analysis (alleles frequencies)				1000 genomes (alleles frequencies)	
		Case		Control			
		C	T	C	T	C	T
rs2569190 (C-260T)	East Asian	0.43	0.57	0.5	0.5	0.43(EAS)	0.57(EAS)
	European	0.51	0.49	0.53	0.47	0.51(EUR)	0.49(EUR)
	America	0.53	0.47	0.53	0.47	0.47(AMR)	0.53(AMR)
	All	0.51	0.49	0.52	0.48	0.53	0.47
C-159T	East Asian	0.44	0.56	0.47	0.53	NA	NA
	European	0.53	0.47	0.52	0.48	NA	NA
	Indian	0.49	0.51	0.44	0.46	NA	NA
	All	0.49	0.51	0.5	0.5	NA	NA

*NA* Not Available, *EAS* East Asian ancestry, *EUR* European ancestry, *AMR* Admixed American, *All* overall individuals from Phase 3 of the 1000 Genomes Project

#### Publication bias and sensitivity analysis

**Fig. 3:**
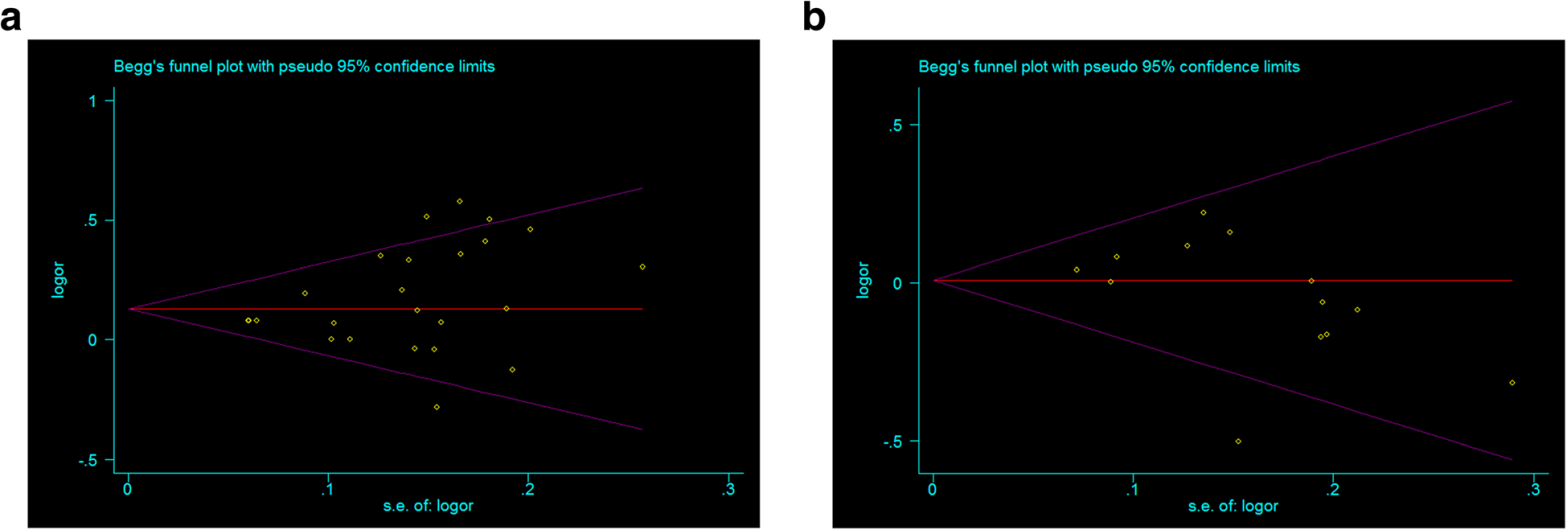
Begg’s funnel plot of publication bias in the meta-analysis of the association of CD14 polymorphisms with CVD risk. **a** rs2569190 and CVD (T vs. C). **b** C-159T and CVD (T vs. C)

**Fig. 4:**
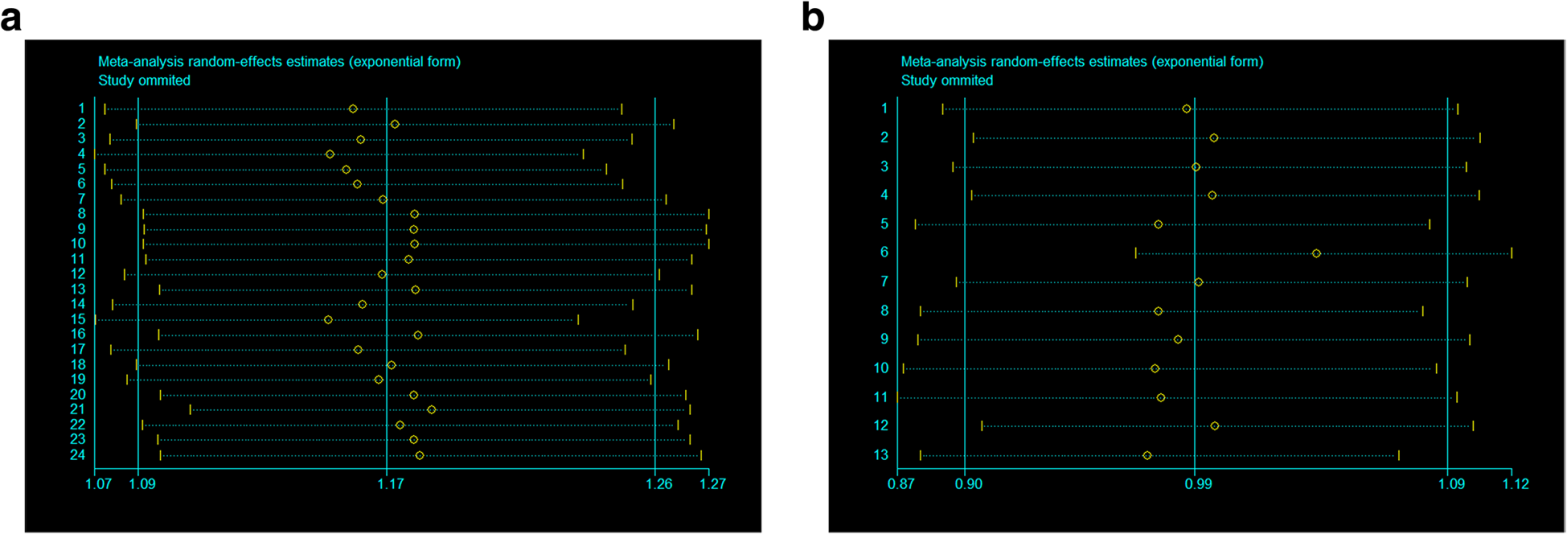
Sensitivity analysis to assess the stability of the meta-analysis. **a** rs2569190 and CVD (T vs. C). **b** C-159T and CVD (T vs. C)

Begg’s funnel plot and Egger’s test were performed to estimate publication bias (Fig. 3a-b). There were no evidence of publication bias for SNP rs2569190 (*p* = 0.118) and C-159T (*p* = 0.077) under allele genetic model (Additional file 1: Table S1; Fig. 3a-b; Additional file 1: Figure S2 a-d). In addition, no significant difference in the Egger’s test neither, suggesting no obvious bias of publication in the present meta-analysis. We also conducted sensitivity analysis to assess the influence of individual studies on the pooled ORs. We found the pooled OR was not substantially altered, when a single study involved in the meta-analysis was deleted each time (Fig. 4a-b; Additional file 1: Figure S3 a-d).

## Discussion

In the present study, we conducted a meta-analysis to evaluate the association between rs2569190 and C-159T with the susceptibility of cardiovascular disease (CVD). It was verified that the T allele of rs2569190 apparently increased the risk of CVD, particularly in East Asian population. In stratified analysis by endpoints, rs2569190 was significantly associated with CHD, whereas, no any allele-specific or genotype-specific relation of the C-159T with cardiovascular disease was observed in diverse ethnicities.

The SNP rs2569190, locating in the promoter region of the *CD14* gene was considered modulating the capacity to stimulate inflammation through the regulation of *CD14* gene expression and plasma soluble CD14 (sCD14) levels [56]. A potential functional role of rs2569190 on CD14 has been suggested as it alters a Sp1 transcription factor binding site and modulates the activity of promoter, the T allele was associated with higher transcription. This SNP was identified to be associated with multiple inflammatory diseases [57, 58]. In previous studies, rs2569190 was indicated to be associated with inflammatory bowel disease [59] and eczema [60]; afterward, studies showed the relevance between rs2569190 and diverse vascular events, such as atherosclerosis [61]. In 1999, rs2569190 was first shown to influence the interaction between CD14 receptor and human coronary artery atherosclerosis and complications [5].

Recently, in the genome-wide association studies (GWAS) of Alex P et al. [62], a robust result was discovered that rs2569190 was significantly associated with CAD in older European-American adults and a potential association in black (European Americans *P* = 6.15e-08, Blacks *P* = 0.04), meanwhile the same findings were also obtained by other investigators in another European population [50]. To check this association in different ethnicities, we carried out a comprehensive ethnicity-specific meta-analysis involving 9413 cases and 7337 controls with 24 separate comparisons and used subgroup analysis as well as the random effect model to deal with the heterogeneity. We found that the association between rs2569190 and CAD achieved significant level in overall population and reached genome-wide significance in East Asian population (T vs. C, OR = 1.370, 95% CI: 1.226–1.531, *P* = 2.86 × 10^− 8^) as well as a certain relevance in the European, but no association in America population. Similarly, in the previous meta-analysis with 28 case–control articles testified a significant association between rs2569190 with CHD in East Asian, but they acquired a negative result in European populations under any genetic models [37], which was associated result in our study. After carefully read, we found that the data in their study were not convinced. Some studies about C-159T were included by researchers [20, 21, 54], and three studies with data deviated from HWE [17, 35, 36]. Papers of this kind were not supposed to pass the inclusion criteria of meta-analysis may deviate from the true results. Therefore, we excluded these studies to carry out a correct meta-analysis.

In our meta-analysis with rs2569190, 16 studies for the European were included. After analyzed, the marginal significance was discovered in European subgroup (*P* = 0.015), which was negative result in another meta-analysis study [37]. It was easy for us to see that the frequencies of the T allele in our European population approximate to that in the overall population. Meanwhile, the T allele of control was discordant with C allele in European and C allele frequency was distinctly larger than T. Besides, we could notice that the alleles frequencies are concordant in the comparison of European subgroup to another meta-analysis study [37]. In addition, we made a comparison for rs2569190 to1000 Genomes Project and found the allele frequencies of rs2569190 in this meta-analysis were consistent with the allele frequencies in the 1000 Genome Project EUR (European ancestry), EAS (East Asian ancestry), respectively, however, there was distinction between the allele frequencies in AMR (Admixed American) and 1000 Genomes Project due to the weak number of studies about American population. More studies with statistical enough power for American race are needed for deeply evaluation.

Of course, so far some research had reported that there was no association was found between rs2569190 and CVD in European population [16, 46], which was contradictory with our findings. It’s normal that such distinct consequences were obtained in separated studies. CVD is considered to be a common multifactor syndrome due to its complicated pathogenesis [63]. A part of previous studies verified that the rs2569190 was associated with CVD in European population [50] and the diametrical results were also carried out in other participants [46]. It was validated that the BMI and smoking will significantly contribute to susceptibility and development of CVD [64]. In addition, the gender difference was the key role in CVD morbidity [65]. However, the basic data which lack of BMI level in participants might lead to inconsistent results. These phenomena and discrepancy needed further investigation on the basis of large sample size.

C-159T base at − 159 lies 49 bp adjacent to an experimentally detected binding site for transcription factor Sp1 at − 110 and 1 bp adjacent to a putative Ap2 site at − 158, it was considered to be an important CD14-activating mediator of inflammatory responses that may result in atherosclerosis, coronary heart disease (CAD), thrombus formation and myocardial infarction (MI) [54]. Previous studies had confirmed that C-159T polymorphism was associated with CAD and indicated that C-159T expression was obviously higher in CAD cases than in controls [19, 23], but there were distinct conflictions among different researchers in the different ethnics [53].

In 2015, Li et al. [38] performed a meta-analysis included 2798 cases and 1669 controls from 7 articles. The results indicated that C-159T could increase the risk of CAD under allelic model (*p* = 0.05), recessive (*p* = 0.01) in the whole population with marginal significance, no significant association was found between them under dominant (*p* = 0.10). A significant association was detected in Chinese population (*p* < 0.05), while there was no significant association in the European subgroup (*p* > 0.05). To precisely explore the association between C-159T and CAD, we analyzed the data that were consistent with HWE. Ultimately, we carried out a comprehensive meta-analysis with 7665 participants. In contrast, we obtained the ineffective relationship between C-159T with any phenotypes of CVD in all subgroups from our study. Then, we compared to previous meta-analysis, and we found that they only included 5 articles about Chinese and 2 studies about European, such deficient data sets may lead to imprecise results. Simultaneously, we realized that the marginal significance under the allelic model and recessive model will be leaded by limited data sizes for C-159T, whereas our study included 13 researches to perform a more comprehensive study which will be more persuasive. In addition, the significant heterogeneity was easily found under all models in their study, of which the heterogeneity reached threshold (*I*^2^ = 83%) in the most relative recessive model. So many limitations were discovered, which did not emerge in our study, will produce contradictory conclusion.

Although no significant association between C-159T and CAD was found in our study, the positive results were discovered by previous studies [22]. We found that the participants which were included by the positive study, were stratified in the gender and the males were much higher than females [19]. Besides, the age was inconsistent in diverse studies, the youngest cohort is 47.9 ± 5.8 and the oldest is 65.30 ± 10.53 in cases of CAD [21, 36]. It’s likely that such differences may, at less in part, attributed to the conclusion difference.

In addition, *CD14* gene, located in 5q23–31, spans 3.9 kb, which encodes the glycoprotein with 375 amino acids [66]. The two identified SNPs, rs2569190, in upstream of the *CD14* promoter at base pair − 260 from the major transcription start site and C-159T, in the 5′ flanking region of the *CD14* gene at position − 159 [67–69]. Many previous studies had suggested that the two polymorphisms will increase sCD14 levels in homozygous carriers of T allele [19, 50] and the base cytosine (C) is replaced by thymine (T) in *CD14* gene polymorphisms rs2569190 and C-159T has been reported being associated with a higher risk of CAD [22]. Thus, the relationship that C-159T whether dependent on rs2569190 to alter the activity of *CD14* gene promoter affect *CD14* gene expression and lead to atherosclerosis, increase the risk of CAD, was not to be researched.

Although we revealed some new discoveries in this study, there were still several limitations should be taken into consideration. In our study, the overall sample size is large, but the size of each study is relatively small, the smallest sample is 54 cases and 70 controls [47], and we need numerous data to validate the relationship between rs2569190 and CVD for further study in American and other populations. For C-159T, the included data in current meta-analysis for ethnicity more from population with European and East Asian origin, and the findings are applicable to only these populations, more studies are required in Indian and other populations. Secondly, we had to indicate that significant between-study heterogeneity that was detected. We used the random effect model to deal with the heterogeneity, but this might induce an imprecise statistic because fixed effect model and random effect model address different research questions. Additionally, we are unable to analyze the actual impact of immanent factors on cardiovascular disease because of the incomplete data. Age is a powerful predictor of cardiovascular adverse events. It is vague why older patients continue to have poor outcomes after ACS despite improved access to contemporary treatment [70]. Meanwhile, the cardiovascular morbidity will also vary due to gender differences [71]. In this study, we did not investigate the contribution of age and gender to the onset of cardiovascular disease due to the insufficient data. Furthermore, the mechanism of CVD is considered to be comprehensive, including gene-gene and gene-environment interactions. To sum up, more studies with enough statistical power are needed for deeply evaluation.

## Conclusions

We conducted a meta-analysis to evaluate the effects of CD14 polymorphisms (rs2569190 and C-159T) on the risk of cardiovascular disease. This meta-analysis indicated that SNP rs2569190 significantly contribute to susceptibility and development of CVD, particularly in the East Asian population and in the subtype CHD group, in addithon, a potential association was observed in the AMI group, T allele acts as a risk factor for cardiovascular disease. However, we failed to acquire the positive association between rs2569190 and other subtypes of CVD. Meanwhile, the associations between C-159T polymorphism with CVD were not observed under any model. Further efforts should be put on investigating the association between the functional mutations within CD14 gene and CVD, and the interactions in potential gene-gene and gene-environment should be comprehensively analyzed.

## Additional file

Additional file 1: **Table S1.** The information of publication bias in all Polymorphisms. **Figure S1.** Forest plot for the meta-analysis of the association between CD14 gene polymorphisms and CVD under the Dominant and Recessive models**. Figure S2.** Begg’s funnel plot of publication bias in the meta-analysis of the association of CD14 polymorphisms with CVD risk**. Figure S3.** Sensitivity analysis to assess the stability of the meta-analysis. (PDF 576 kb)

## Abbreviations

95% CI: 95% confidence interval
AMI: Acute myocardial infarction
AMR: Admixed American
CC: Case–control
CHD: Coronary heart disease
CVD: Cardiovascular disease
EAS: East Asian ancestry
EH –LVH/EH –LVH: Essential hypertension with left/not left ventricular hypertrophy
EUR: European ancestry
HWE: Hardy-Weinberg Equilibrium
MI: Myocardial infarction
NA: Not available
NCC: Nested case–control
OR: Odd ratio
