# Supplementary material for: Effect of CD14 polymorphisms on the risk of cardiovascular disease: evidence from a meta-analysis
Source: Lipids Health Dis. 2019 Mar 28;18:74. doi: 10.1186/s12944-019-1018-3 (PMC6439994; doi:10.1186/s12944-019-1018-3)
Supplement: Supplementary file 1 — Table S1. The information of publication bias in all Polymorphisms. Figure S1. Forest plot for the meta-analysis of the association between CD14 gene polymorphisms and CVD under the Dominant and Recessive models. Figure S2. Begg’s funnel plot of publication bias in the meta-analysis of the association of CD14 polymorphisms with CVD risk. Figure S3. Sensitivity analysis to assess the stability of the meta-analysis. (PDF 576 kb) [file 12944_2019_1018_MOESM1_ESM.pdf]

Effect of CD14 polymorphisms on the Risk of Cardiovascular Disease: Evidence from a Meta-Analysis

Running head: CD14 polymorphisms and Cardiovascular

Jin-Jian Xu[1,2#], Ke-Qi Liu[3#], Zhi-Min Ying[4#], Xiao-Wei Zhu[1], Xue-Jin Xu[2], Pian-Pian Zhao[1], Wei-Yang Bai[1], Mo-Chang Qiu[3], Pei-Kuan Cong[2], Xing-Wei Zhang[2], Hou-Feng Zheng[1,2*]

1, Diseases & Population (DaP) Geninfo Lab, School of Life Sciences, Westlake University and Westlake Institute for Advanced Study, Hangzhou, Zhejiang, China.

2, Institute of Ageing Research and the Affiliated Hospital, School of Medicine, Hangzhou Normal University, Hangzhou, Zhejiang, China

3, Jiangxi Medical College, Shangrao, Jiangxi, China

4, The Second Affiliated Hospital, Zhejiang University School of Medicine, Hangzhou, Zhejiang, China

*Corresponding to: Hou-Feng Zheng, MD, PhD.

E-mail: zhenghoufeng@wias.org.cn

Table S1. The information of publication bias in all Polymorphisms

Polymorphisms	Allele model		Dominant model		Recessive model	
	Z	P	Z	P	Z	P
C-159T	1.77	0.077	1.4	0.161	1.4	0.161
C-260T	1.56	0.118	2.51	0.012	1.12	0.264

a1

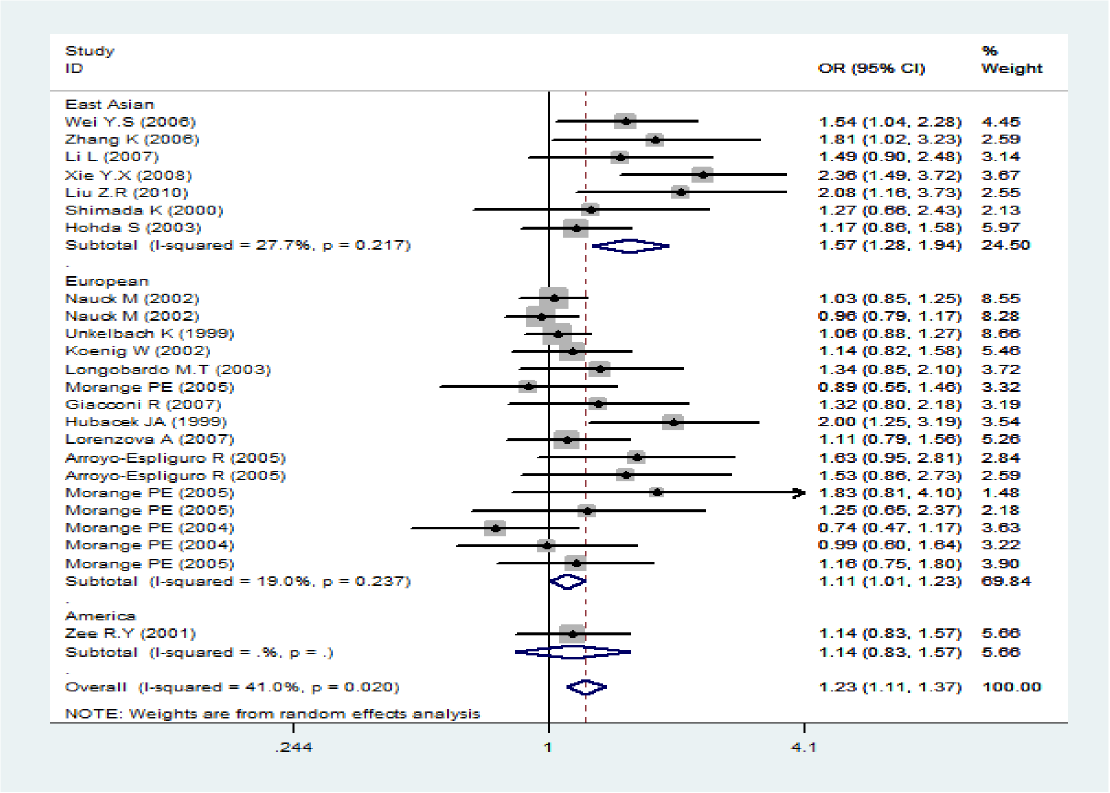

a2

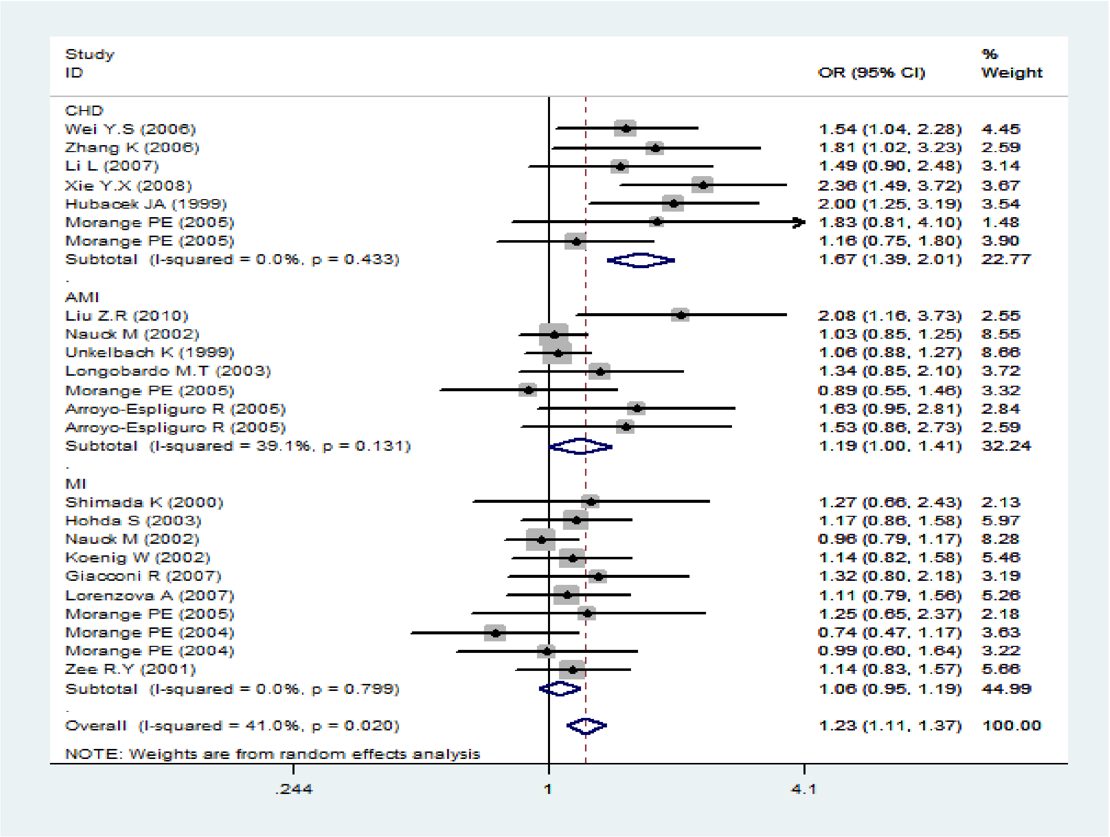

Study ID
OR (95% CI)
% Weight
CHD
Wei Y.S (2006) 1.54 (1.04, 2.28) 4.45
Zhang K (2006) 1.81 (1.02, 3.23) 2.59
Li L (2007) 1.49 (0.90, 2.48) 3.14
Xie Y.X (2008) 2.36 (1.49, 3.72) 3.67
Hubacek JA (1999) 2.00 (1.25, 3.19) 3.54
Morange PE (2005) 1.83 (0.81, 4.10) 1.48
Morange PE (2005) 1.16 (0.75, 1.80) 3.90
Subtotal (I-squared = 0.0%, p = 0.433) 1.67 (1.39, 2.01) 22.77
.
AMI
Liu Z.R (2010) 2.08 (1.16, 3.73) 2.55
Nauck M (2002) 1.03 (0.85, 1.25) 8.55
Unkelbach K (1999) 1.06 (0.88, 1.27) 8.66
Longobardo M.T (2003) 1.34 (0.85, 2.10) 3.72
Morange PE (2005) 0.89 (0.55, 1.46) 3.32
Arroyo-Espliguro R (2005) 1.63 (0.95, 2.81) 2.84
Arroyo-Espliguro R (2005) 1.53 (0.86, 2.73) 2.59
Subtotal (I-squared = 39.1%, p = 0.131) 1.19 (1.00, 1.41) 32.24
.
MI
Shimada K (2000) 1.27 (0.66, 2.43) 2.13
Hohda S (2003) 1.17 (0.86, 1.58) 5.97
Nauck M (2002) 0.96 (0.79, 1.17) 8.28
Koenig W (2002) 1.14 (0.82, 1.58) 5.46
Giacconi R (2007) 1.32 (0.80, 2.18) 3.19
Lorenzova A (2007) 1.11 (0.79, 1.56) 5.26
Morange PE (2005) 1.25 (0.65, 2.37) 2.18
Morange PE (2004) 0.74 (0.47, 1.17) 3.63
Morange PE (2004) 0.99 (0.60, 1.64) 3.22
Zee R.Y (2001) 1.14 (0.83, 1.57) 5.66
Subtotal (I-squared = 0.0%, p = 0.799) 1.06 (0.95, 1.19) 44.99
.
Overall (I-squared = 41.0%, p = 0.020) 1.23 (1.11, 1.37) 100.00
NOTE: Weights are from random effects analysis
.244
1
4.1

b1

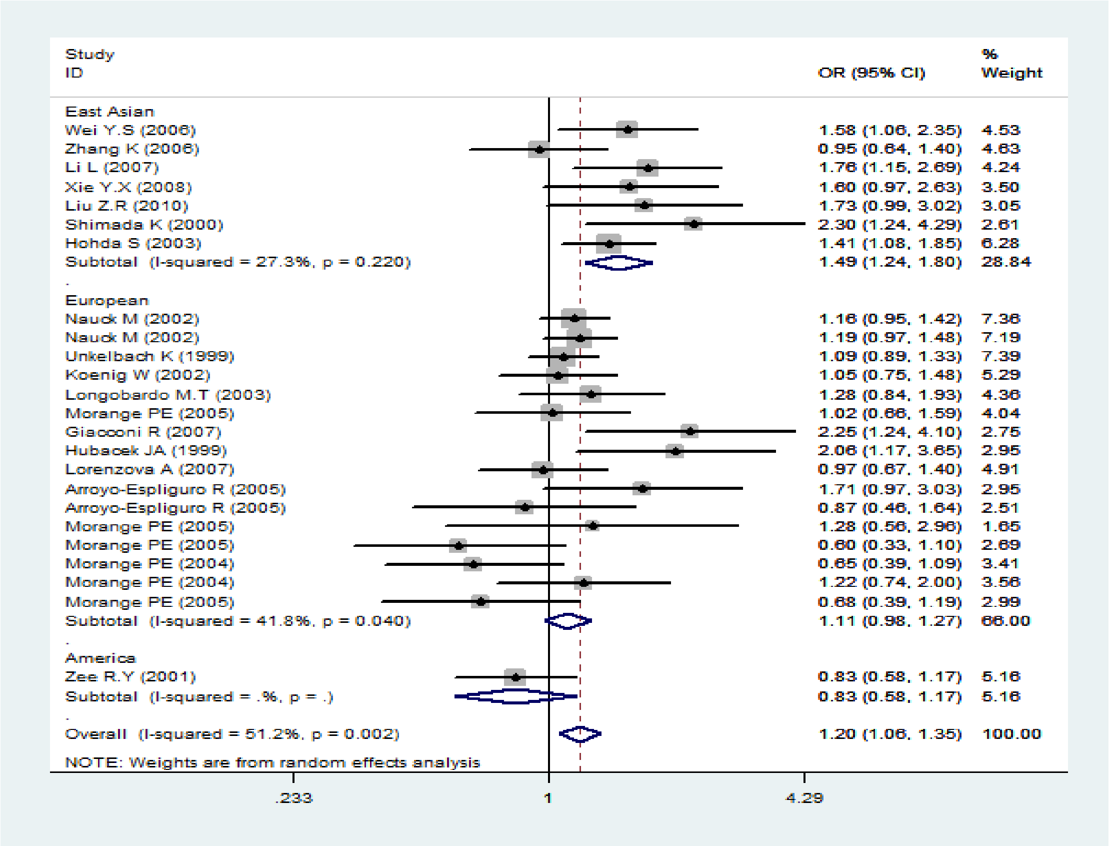

Study ID
OR (95% CI)
% Weight
East Asian
Wei Y.S (2006) 1.58 (1.06, 2.35) 4.53
Zhang K (2006) 0.95 (0.64, 1.40) 4.63
Li L (2007) 1.76 (1.15, 2.69) 4.24
Xie Y.X (2008) 1.60 (0.97, 2.63) 3.50
Liu Z.R (2010) 1.73 (0.99, 3.02) 3.05
Shimada K (2000) 2.30 (1.24, 4.29) 2.61
Hohda S (2003) 1.41 (1.08, 1.85) 6.28
Subtotal (I-squared = 27.3%, p = 0.220) 1.49 (1.24, 1.80) 28.84
.
European
Nauck M (2002) 1.16 (0.95, 1.42) 7.36
Nauck M (2002) 1.19 (0.97, 1.48) 7.19
Unkelbach K (1999) 1.09 (0.89, 1.33) 7.39
Koenig W (2002) 1.05 (0.75, 1.48) 5.29
Longobardo M.T (2003) 1.28 (0.84, 1.93) 4.36
Morange PE (2005) 1.02 (0.66, 1.59) 4.04
Giacconi R (2007) 2.25 (1.24, 4.10) 2.75
Hubacek JA (1999) 2.06 (1.17, 3.65) 2.95
Lorenzova A (2007) 0.97 (0.67, 1.40) 4.91
Arroyo-Espliguro R (2005) 1.71 (0.97, 3.03) 2.95
Arroyo-Espliguro R (2005) 0.87 (0.46, 1.64) 2.51
Morange PE (2005) 1.28 (0.56, 2.96) 1.65
Morange PE (2005) 0.60 (0.33, 1.10) 2.69
Morange PE (2004) 0.65 (0.39, 1.09) 3.41
Morange PE (2004) 1.22 (0.74, 2.00) 3.56
Morange PE (2005) 0.68 (0.39, 1.19) 2.99
Subtotal (I-squared = 41.8%, p = 0.040) 1.11 (0.98, 1.27) 66.00
.
America
Zee R.Y (2001) 0.83 (0.58, 1.17) 5.16
Subtotal (I-squared = .%, p = .) 0.83 (0.58, 1.17) 5.16
.
Overall (I-squared = 51.2%, p = 0.002) 1.20 (1.06, 1.35) 100.00
NOTE: Weights are from random effects analysis
.233
1
4.29

b2

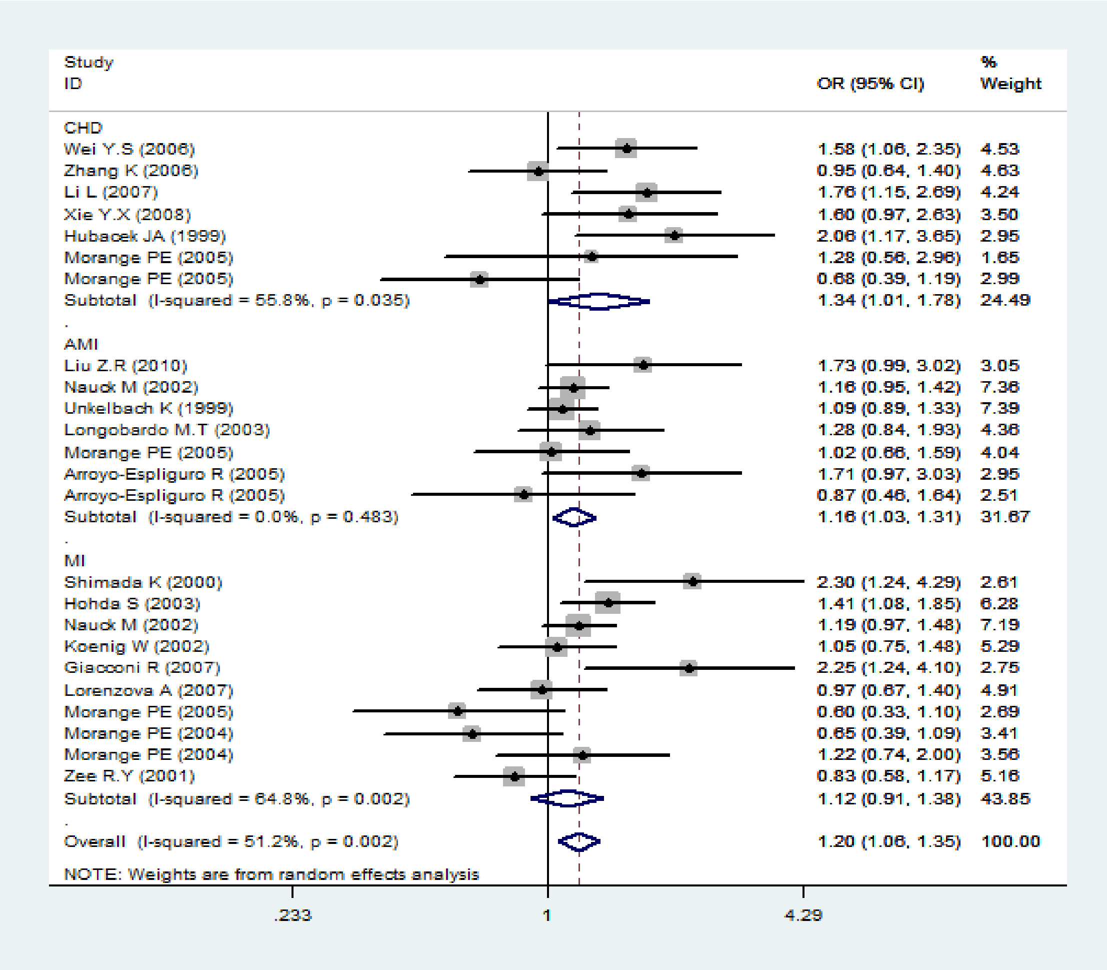
Study
ID
OR (95% CI)
%
Weight
CHD
Wei Y.S (2006) 1.58 (1.06, 2.35) 4.53
Zhang K (2006) 0.95 (0.64, 1.40) 4.63
Li L (2007) 1.76 (1.15, 2.69) 4.24
Xie Y.X (2008) 1.60 (0.97, 2.63) 3.50
Hubacek JA (1999) 2.06 (1.17, 3.65) 2.95
Morange PE (2005) 1.28 (0.56, 2.96) 1.65
Morange PE (2005) 0.68 (0.39, 1.19) 2.99
Subtotal (I-squared = 55.8%, p = 0.035) 1.34 (1.01, 1.78) 24.49
AMI
Liu Z.R (2010) 1.73 (0.99, 3.02) 3.05
Nauck M (2002) 1.16 (0.95, 1.42) 7.36
Unkelbach K (1999) 1.09 (0.89, 1.33) 7.39
Longobardo M.T (2003) 1.28 (0.84, 1.93) 4.36
Morange PE (2005) 1.02 (0.66, 1.59) 4.04
Arroyo-Espliguro R (2005) 1.71 (0.97, 3.03) 2.95
Arroyo-Espliguro R (2005) 0.87 (0.46, 1.64) 2.51
Subtotal (I-squared = 0.0%, p = 0.483) 1.16 (1.03, 1.31) 31.67
MI
Shimada K (2000) 2.30 (1.24, 4.29) 2.61
Hohda S (2003) 1.41 (1.08, 1.85) 6.28
Nauck M (2002) 1.19 (0.97, 1.48) 7.19
Koenig W (2002) 1.05 (0.75, 1.48) 5.29
Giacconi R (2007) 2.25 (1.24, 4.10) 2.75
Lorenzova A (2007) 0.97 (0.67, 1.40) 4.91
Morange PE (2005) 0.60 (0.33, 1.10) 2.69
Morange PE (2004) 0.65 (0.39, 1.09) 3.41
Morange PE (2004) 1.22 (0.74, 2.00) 3.56
Zee R.Y (2001) 0.83 (0.58, 1.17) 5.16
Subtotal (I-squared = 64.8%, p = 0.002) 1.12 (0.91, 1.38) 43.85
Overall (I-squared = 51.2%, p = 0.002) 1.20 (1.06, 1.35) 100.00
NOTE: Weights are from random effects analysis
.233
1
4.29

c1

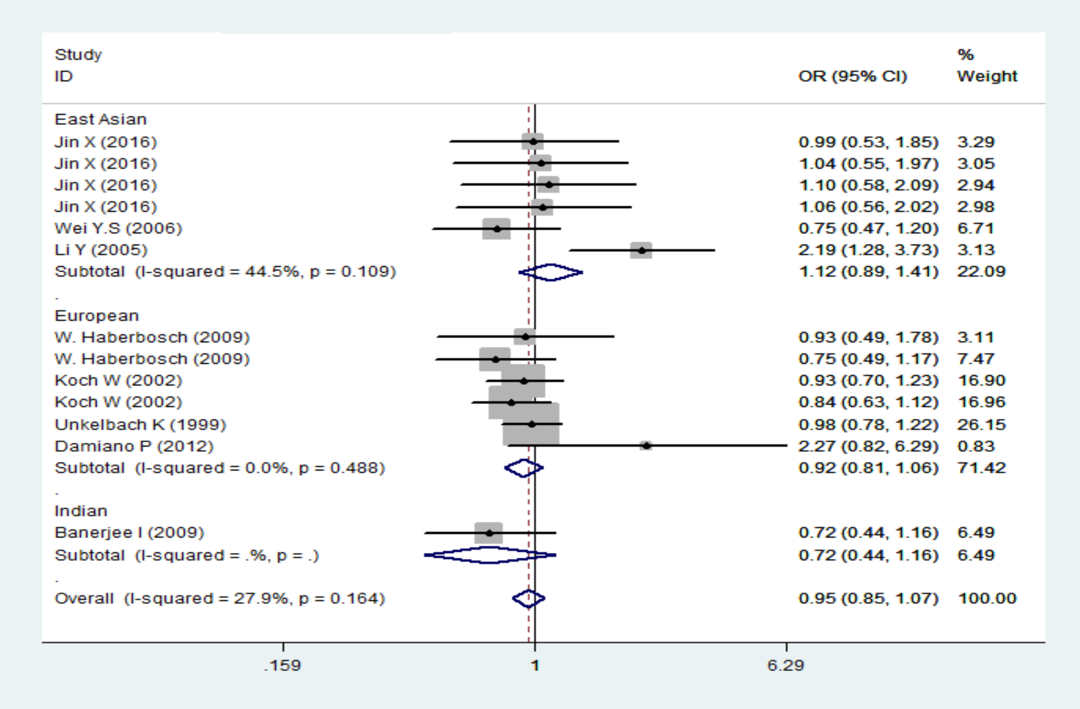
Study
ID
OR (95% CI)
%
Weight
East Asian
Jin X (2016) 0.99 (0.53, 1.85) 3.29
Jin X (2016) 1.04 (0.55, 1.97) 3.05
Jin X (2016) 1.10 (0.58, 2.09) 2.94
Jin X (2016) 1.06 (0.56, 2.02) 2.98
Wei Y.S (2006) 0.75 (0.47, 1.20) 6.71
Li Y (2005) 2.19 (1.28, 3.73) 3.13
Subtotal (I-squared = 44.5%, p = 0.109) 1.12 (0.89, 1.41) 22.09
European
W. Haberbosch (2009) 0.93 (0.49, 1.78) 3.11
W. Haberbosch (2009) 0.75 (0.49, 1.17) 7.47
Koch W (2002) 0.93 (0.70, 1.23) 16.90
Koch W (2002) 0.84 (0.63, 1.12) 16.96
Unkelbach K (1999) 0.98 (0.78, 1.22) 26.15
Damiano P (2012) 2.27 (0.82, 6.29) 0.83
Subtotal (I-squared = 0.0%, p = 0.488) 0.92 (0.81, 1.06) 71.42
Indian
Banerjee I (2009) 0.72 (0.44, 1.16) 6.49
Subtotal (I-squared = .%, p = .) 0.72 (0.44, 1.16) 6.49
Overall (I-squared = 27.9%, p = 0.164) 0.95 (0.85, 1.07) 100.00
.159
1
6.29

c2

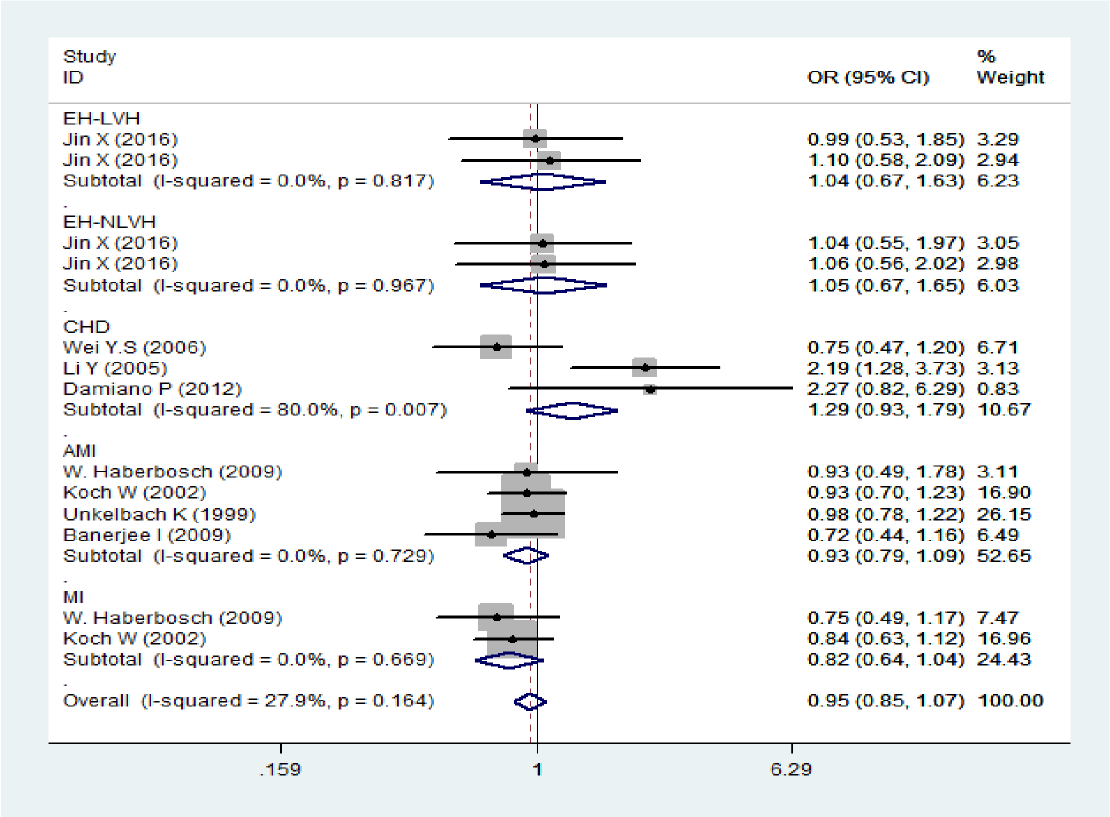
Study
ID
OR (95% CI)
%
Weight
EH-LVH
Jin X (2016) 0.99 (0.53, 1.85) 3.29
Jin X (2016) 1.10 (0.58, 2.09) 2.94
Subtotal (I-squared = 0.0%, p = 0.817) 1.04 (0.67, 1.63) 6.23
EH-NLVH
Jin X (2016) 1.04 (0.55, 1.97) 3.05
Jin X (2016) 1.06 (0.56, 2.02) 2.98
Subtotal (I-squared = 0.0%, p = 0.967) 1.05 (0.67, 1.65) 6.03
CHD
Wei Y.S (2006) 0.75 (0.47, 1.20) 6.71
Li Y (2005) 2.19 (1.28, 3.73) 3.13
Damiano P (2012) 2.27 (0.82, 6.29) 0.83
Subtotal (I-squared = 80.0%, p = 0.007) 1.29 (0.93, 1.79) 10.67
AMI
W. Haberbosch (2009) 0.93 (0.49, 1.78) 3.11
Koch W (2002) 0.93 (0.70, 1.23) 16.90
Unkelbach K (1999) 0.98 (0.78, 1.22) 26.15
Banerjee I (2009) 0.72 (0.44, 1.16) 6.49
Subtotal (I-squared = 0.0%, p = 0.729) 0.93 (0.79, 1.09) 52.65
MI
W. Haberbosch (2009) 0.75 (0.49, 1.17) 7.47
Koch W (2002) 0.84 (0.63, 1.12) 16.96
Subtotal (I-squared = 0.0%, p = 0.669) 0.82 (0.64, 1.04) 24.43
Overall (I-squared = 27.9%, p = 0.164) 0.95 (0.85, 1.07) 100.00
.159
1
6.29

d1

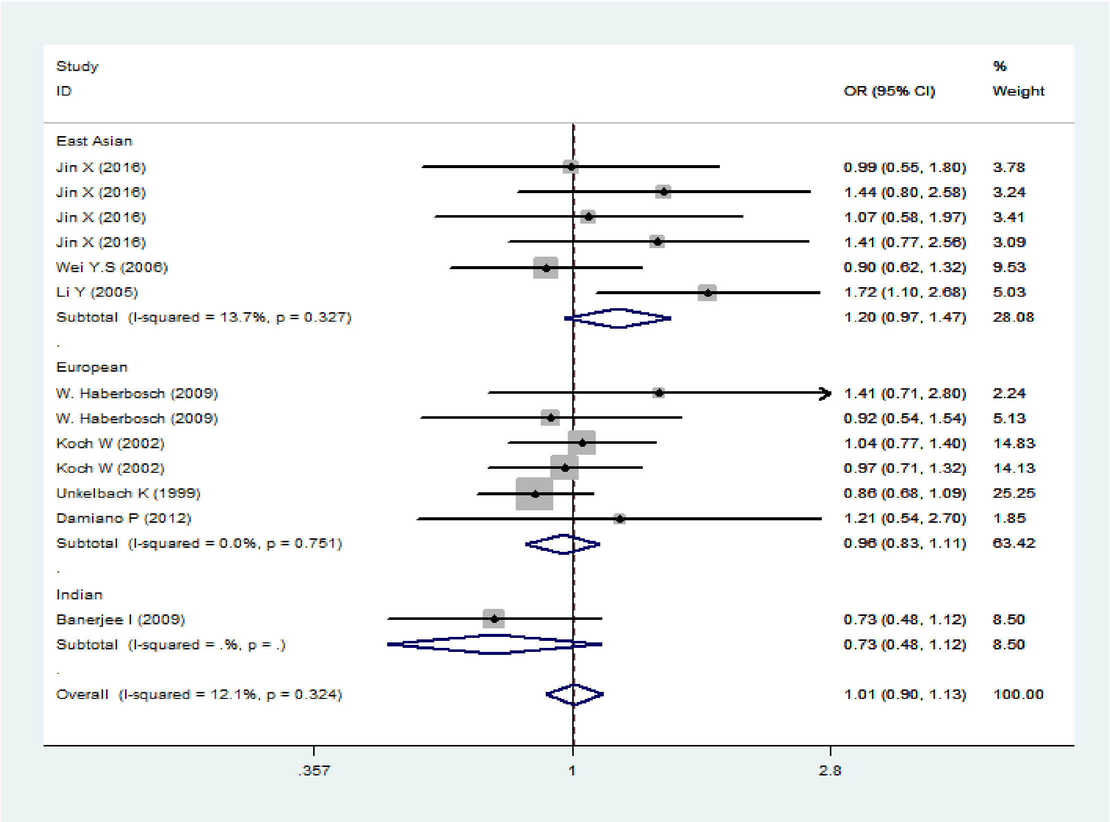
Study
ID
OR (95% CI)
%
Weight
East Asian
Jin X (2016) 0.99 (0.55, 1.80) 3.78
Jin X (2016) 1.44 (0.80, 2.58) 3.24
Jin X (2016) 1.07 (0.58, 1.97) 3.41
Jin X (2016) 1.41 (0.77, 2.56) 3.09
Wei Y.S (2006) 0.90 (0.62, 1.32) 9.53
Li Y (2005) 1.72 (1.10, 2.68) 5.03
Subtotal (I-squared = 13.7%, p = 0.327) 1.20 (0.97, 1.47) 28.08
European
W. Haberbosch (2009) 1.41 (0.71, 2.80) 2.24
W. Haberbosch (2009) 0.92 (0.54, 1.54) 5.13
Koch W (2002) 1.04 (0.77, 1.40) 14.83
Koch W (2002) 0.97 (0.71, 1.32) 14.13
Unkelbach K (1999) 0.86 (0.68, 1.09) 25.25
Damiano P (2012) 1.21 (0.54, 2.70) 1.85
Subtotal (I-squared = 0.0%, p = 0.751) 0.96 (0.83, 1.11) 63.42
Indian
Banerjee I (2009) 0.73 (0.48, 1.12) 8.50
Subtotal (I-squared = .%, p = .) 0.73 (0.48, 1.12) 8.50
Overall (I-squared = 12.1%, p = 0.324) 1.01 (0.90, 1.13) 100.00
.357
1
2.8

d2

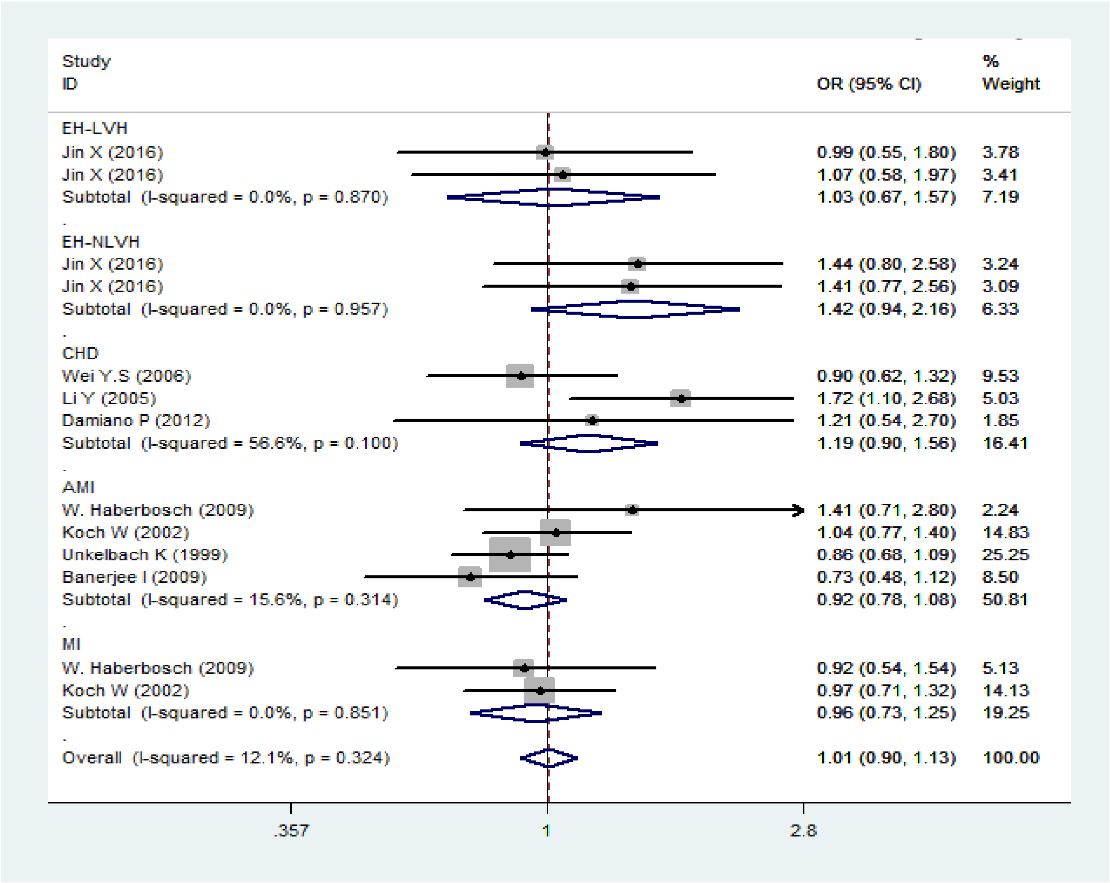

Figure S1. Forest plot for the meta-analysis of the association between CD14 gene polymorphisms and CVD under the Dominant and Recessive models

(a1) rs2569190 and CVD. (CC VS.TT+CT), stratification by ethnicity
(a2) rs2569190 and CVD. (CC VS.TT+CT), stratification by endpoints
(b1) rs2569190 and CVD. (TT VS.CC+CT), stratification by ethnicity
(b2) rs2569190 and CVD. (TT VS.CC+CT), stratification by endpoints
(c1) C-159T and CVD. (CC VS.TT+CT), stratification by ethnicity
(c2) C-159T and CVD. (CC VS.TT+CT), stratification by endpoints
(d1) C-159T and CVD. (TT VS.CC+CT), stratification by ethnicity
(d2) C-159T and CVD. (TT VS.CC+CT), stratification by endpoints

a

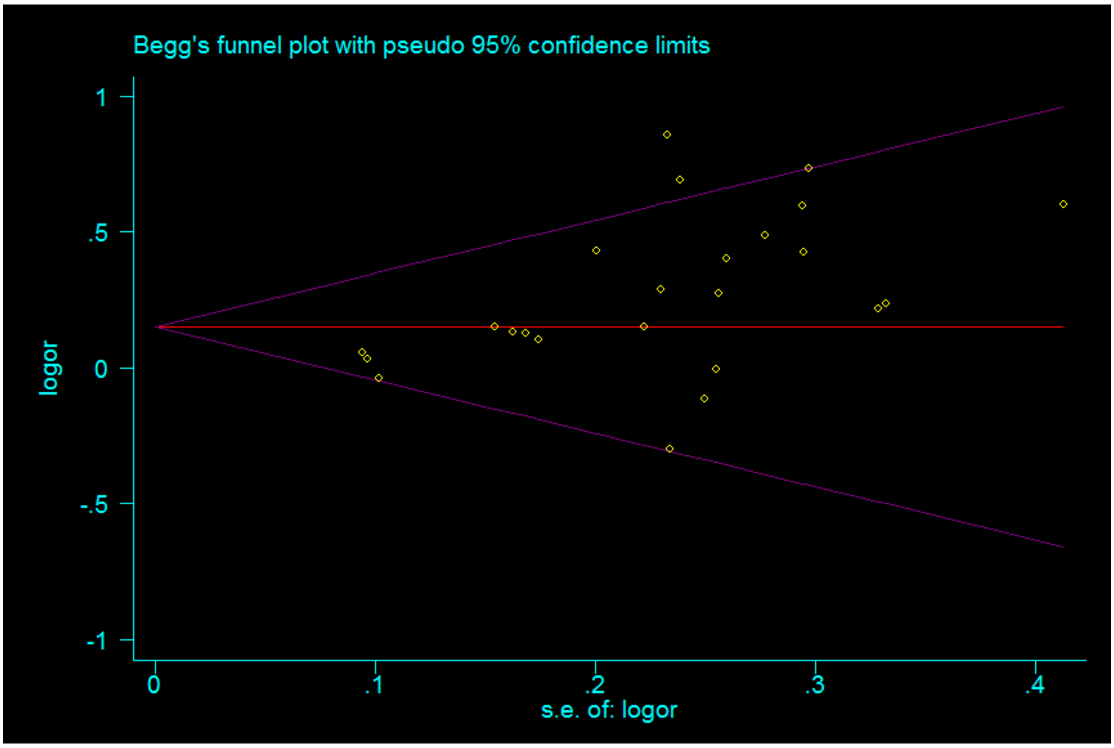

Begg's funnel plot with pseudo 95% confidence limits
1
.5
0
-.5
-1
logor
0
.1
.2
.3
.4
s.e. of: logor

b

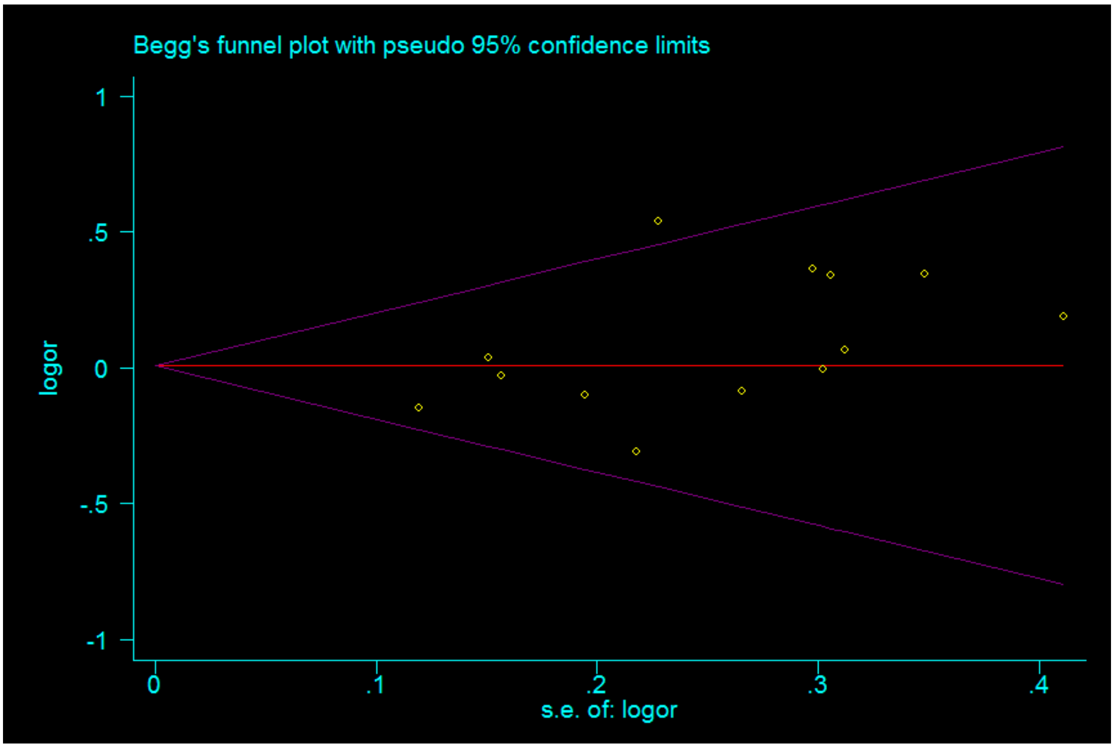

Begg's funnel plot with pseudo 95% confidence limits
1
.5
0
-.5
-1
logor
0
.1
.2
.3
.4
s.e. of: logor

c

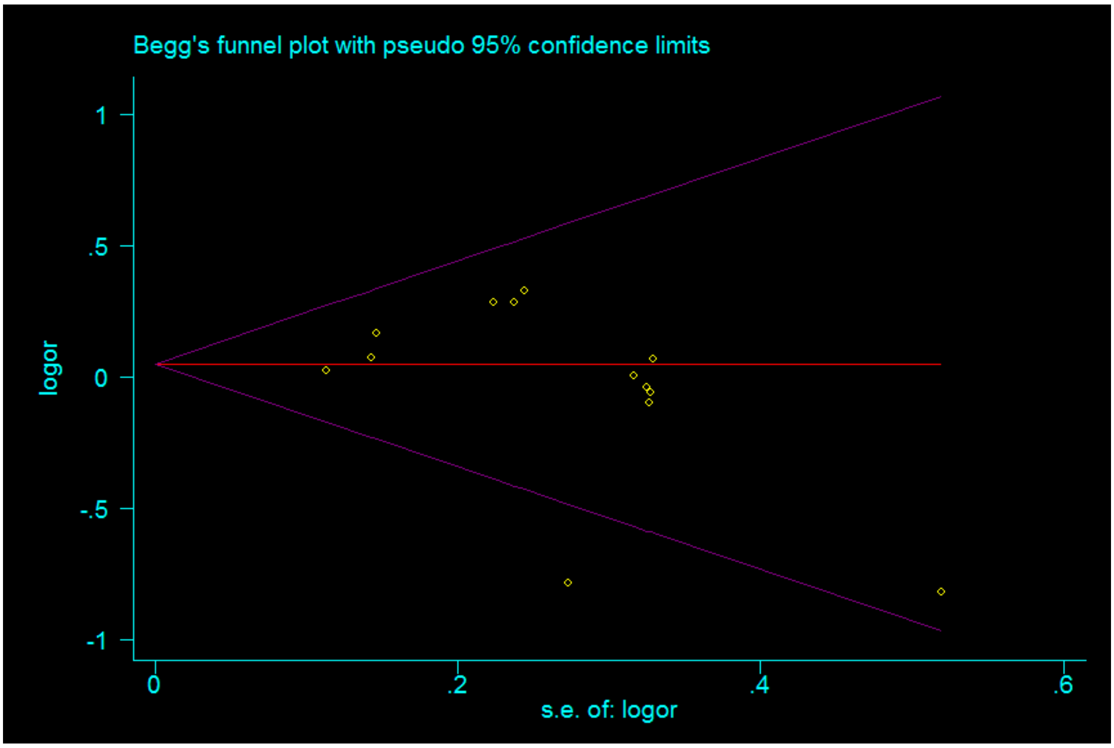
Begg's funnel plot with pseudo 95% confidence limits
1
.5
0
-.5
-1
logor
0
.2
.4
.6
s.e. of: logor

d

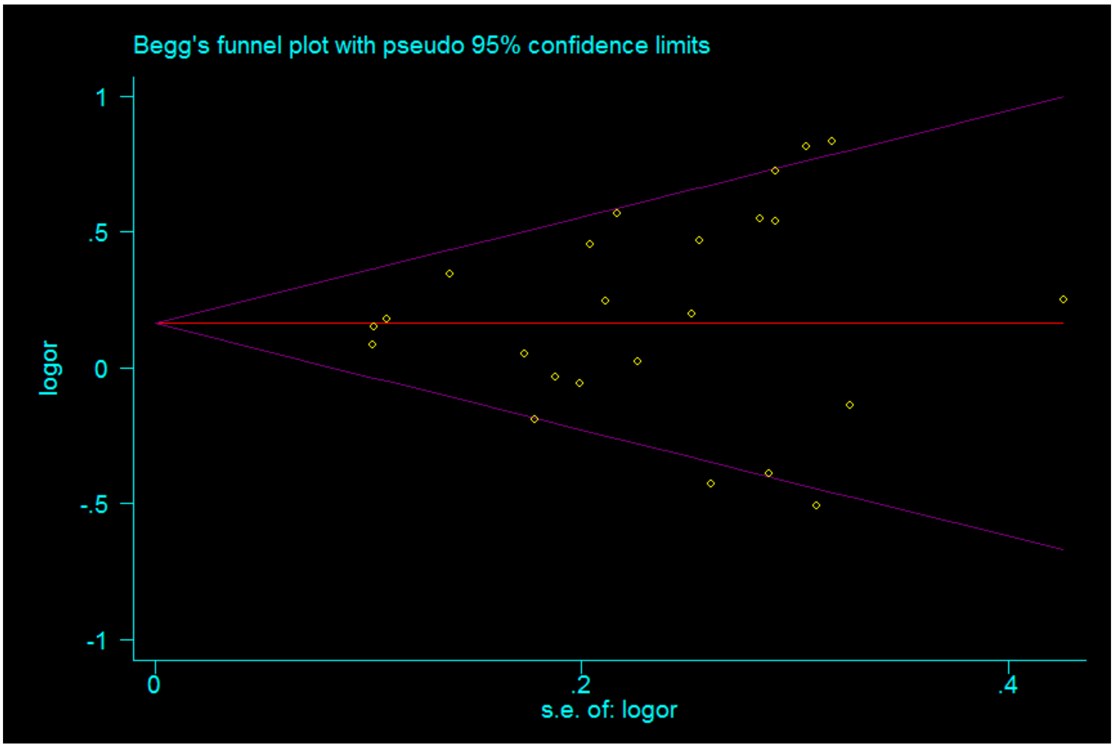
Begg's funnel plot with pseudo 95% confidence limits
1
.5
0
-.5
-1
logor
0
.2
.4
s.e. of: logor

Figure S2. Begg's funnel plot of publication bias in the meta-analysis of the association of CD14 polymorphisms with CVD risk

(a) rs2569190 and CVD. (CC VS.TT+CT)
(b) rs2569190 and CVD. (TT VS.CC+CT)
(c) C-159T and CVD. (CC VS.TT+CT)
(d) C-159T and CVD. (TT VS.CC+CT)

a

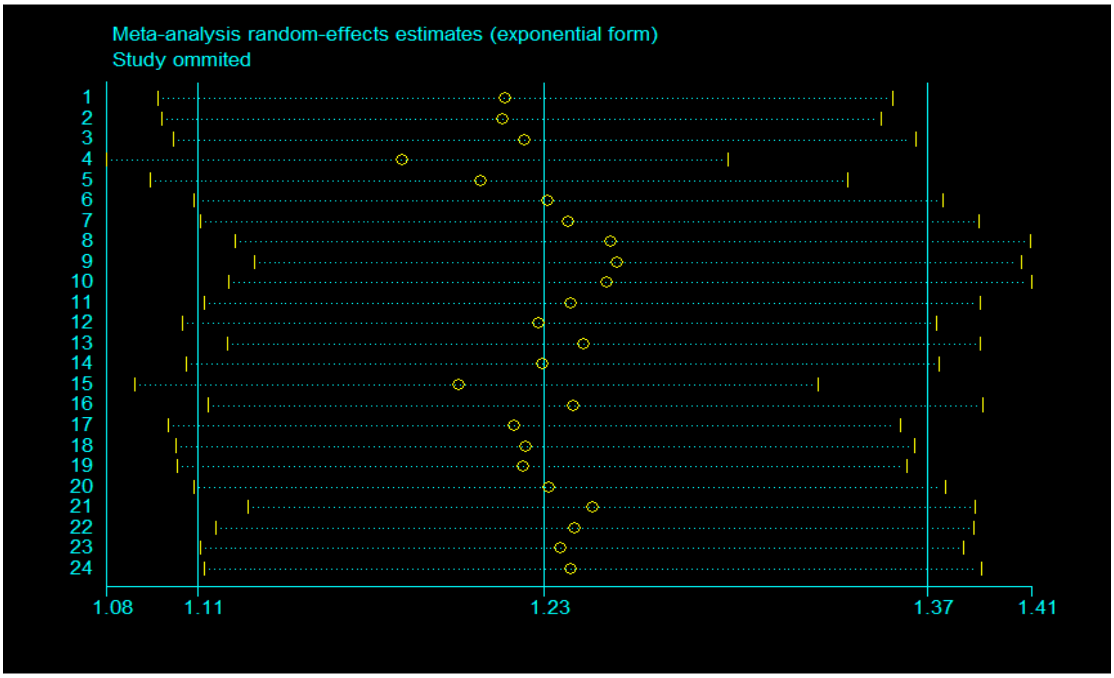

b

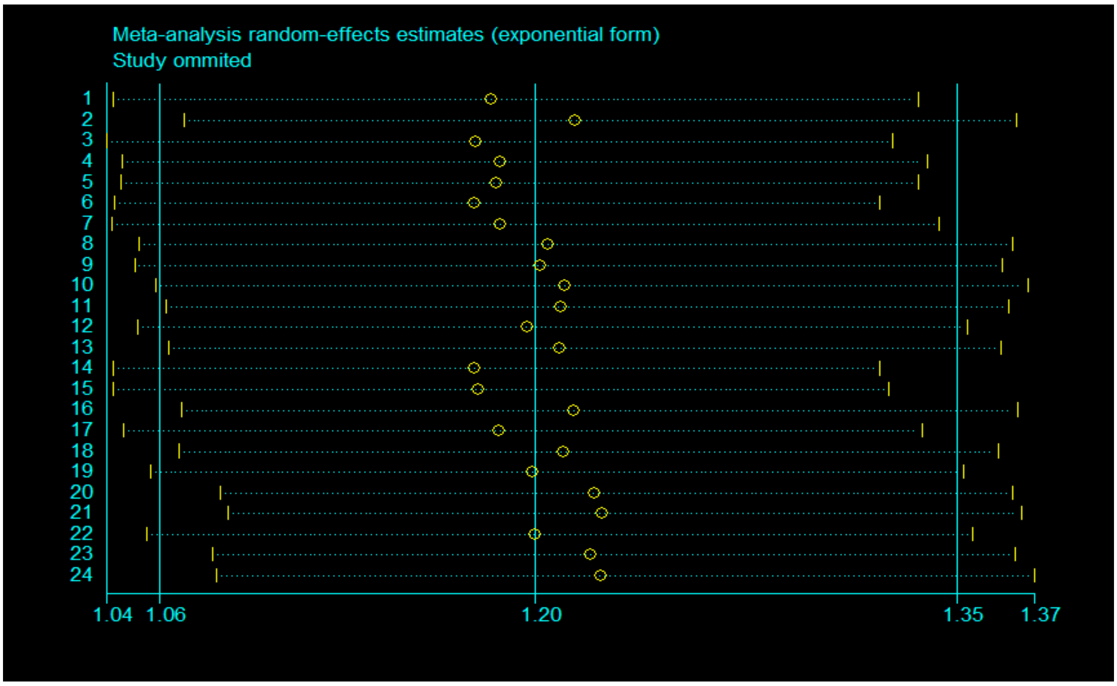

c

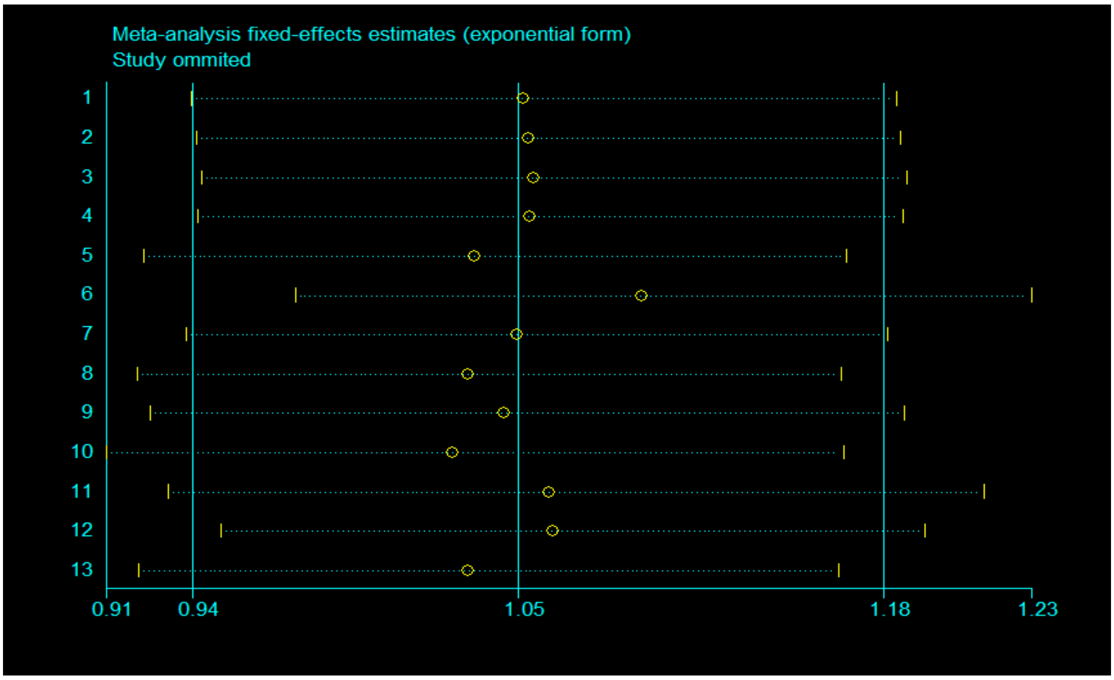

d

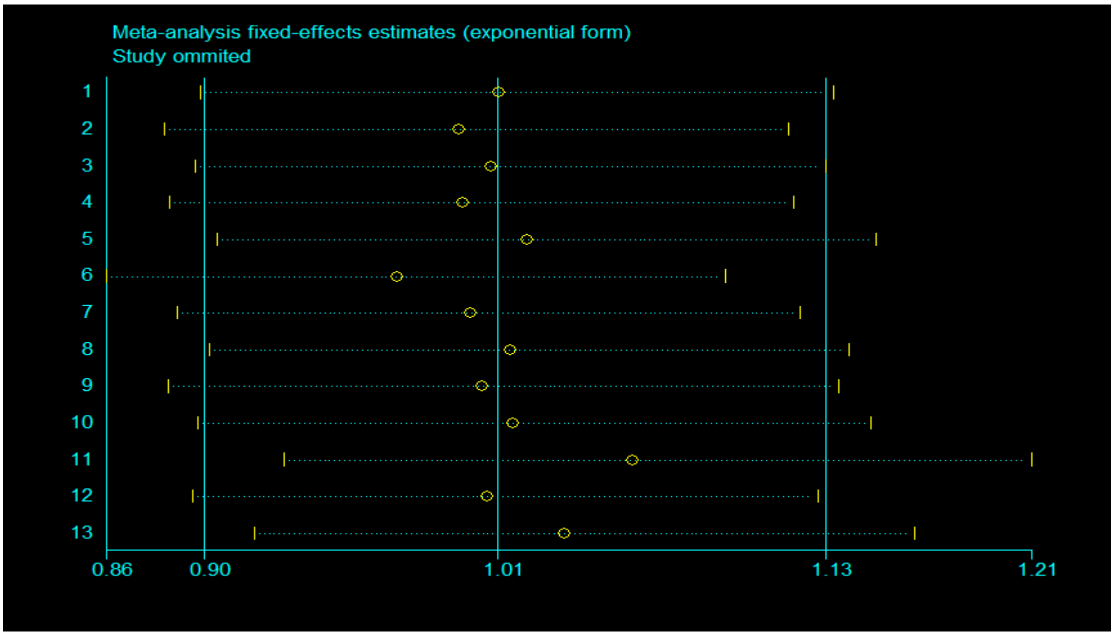

Figure S3. Sensitivity analysis to assess the stability of the meta-analysis

(a) rs2569190 and CVD. (CC VS.TT+CT)

(b) rs2569190 and CVD. (TT VS.CC+CT)

(c) C-159T and CVD. (CC VS.TT+CT)

(d) C-159T and CVD. (TT VS.CC+CT)
